# Range Shifts of the Endangered *Luehdorfia chinensis chinensis* (Lepidoptera, Papilionidae) and Its Specific Hosts in China Under Climate Change

**DOI:** 10.1002/ece3.72057

**Published:** 2025-08-23

**Authors:** Ze Lan, Guangfu Zhang

**Affiliations:** ^1^ Jiangsu Key Laboratory of Biodiversity and Biotechnology, School of Life Sciences Nanjing Normal University Nanjing China

**Keywords:** Biomod2, host choice, *Luehdorfia chinensis chinensis*, niche overlap, suitable range

## Abstract

*Luehdorfia chinensis chinensis*, endemic to China, is an endangered, rare, and protected butterfly with high host specificity. However, little is known about how this butterfly and its host plants respond to climate change. In this study, we built ensemble models in the Biomod2 platform to predict the potential distributions of *L. c. chinensis* and its two host plants, identify key environmental factors, and calculate the niche overlaps between them. The results showed that under the current climate, *L. c. chinensis* covered a suitable area of approximately 1,146,520 km^2^, accounting for 11.95% of China's total territory, and was primarily distributed in central and southeastern China. Precipitation of the driest quarter was identified as the primary factor influencing the distribution of *L. c. chinensis*, whereas precipitation‐related variables were the primary factors influencing the distributions of both host plants. Under future climate scenarios, the butterfly is projected to increase slightly in suitable habitat, while the two hosts show contrasting trends in range shift and niche overlap. Our findings indicate that climate change not only alters the extent and integrity of suitable habitats of the butterfly and its hosts but also affects its larval host choice. Therefore, it is essential to take into account the suitable habitats of the endangered butterfly and its hosts when developing climate‐adapted conservation strategies.

## Graphical abstract

This study forecasted the climate‐driven distribution shifts of China's endangered Luehdorfia chinensis chinensis and its two host plants using Biomod2 ensemble models. Precipitation was identified as the key factor shaping their ranges, with the butterfly's future habitat projected to expand slightly, and the hosts showed divergent shifts and niche overlap changes. Climate change may not only alter the extent and integrity of suitable habitats of the butterfly and its hosts but also affect its larval host choice.

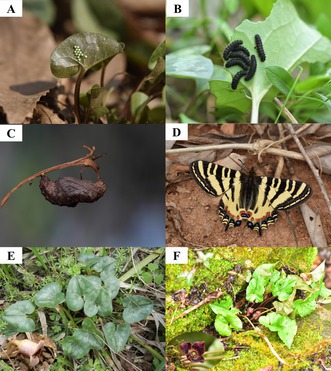

## 1 Introduction

Climate change is profoundly affecting species distribution, genetic diversity, behavior, and adaptive capacity (Lim et al. 2024). As the most diverse and abundant animal group on the earth, insects play a critical role in ecosystem functioning, and their population dynamics exhibit a high sensitivity to climate change (Kellermann and Van Heerwaarden 2019). Existing studies have demonstrated that climate change has a significant impact on both the diversity and abundance of insect species (Forister et al. 2019). One major reason is a rise in temperature resulting from global warming, and this may exceed the tolerance limits of many insects, thereby affecting their survival and reproduction (González‐Tokman et al. 2020). Moreover, there is considerable variation among insect taxa and regions in their responses to climate change (Wagner 2020). Current researches has mainly focused on pollinators and invasive insect species (Desurmont et al. 2014; Pliszko et al. 2025). For instance, Vasiliev and Greenwood (2021) contend that climate change threatens the survival of pollinators through multiple mechanisms, such as reducing habitat range and genetic diversity, disrupting winter weather patterns, and increasing the frequency of extreme events. Zhang et al. (2024) project that the invasive beetle *Anoplophora glabripennis* will expand its suitable habitat toward higher latitudes under global warming (Zhang et al. 2024). In contrast, relatively little attention has been paid to endemic or restricted‐range insects, which generally have small population sizes and limited distributions, making them particularly vulnerable to climate change.

Insects can be categorized by dietary breadth as generalists or specialists. Generalist insects can exploit a wide variety of food sources, which confer greater flexibility and adaptability in the face of climate change. In contrast, specialist insects, which depend exclusively on a narrow range of foods, are less able to shift their diet and may be more vulnerable (Zografou et al. 2021). Consequently, specialist insects may face greater risks under climate change. For native insects, if they are specialists, theoretically, climate change has more effects on them than on generalists. These specialists and their host plants have coevolved over long periods, establishing mutually beneficial interactions (Denno et al. 1995); however, under climate change, both parties are likely to make corresponding shifts in their spatial and temporal distributions. Climate change may therefore disrupt these original interactions. To date, most studies have focused on the insects themselves rather than on their hosts. For example, Hu et al. (2019) indicated that climate change might lead to its range contraction and habitat fragmentation for *Bhutanitis thaidina*, a rare butterfly endemic to China, thereby increasing the butterfiy's risk of extinction. However, little is known about how native insects and their host plants jointly respond to climate change.

**FIGURE 1:**
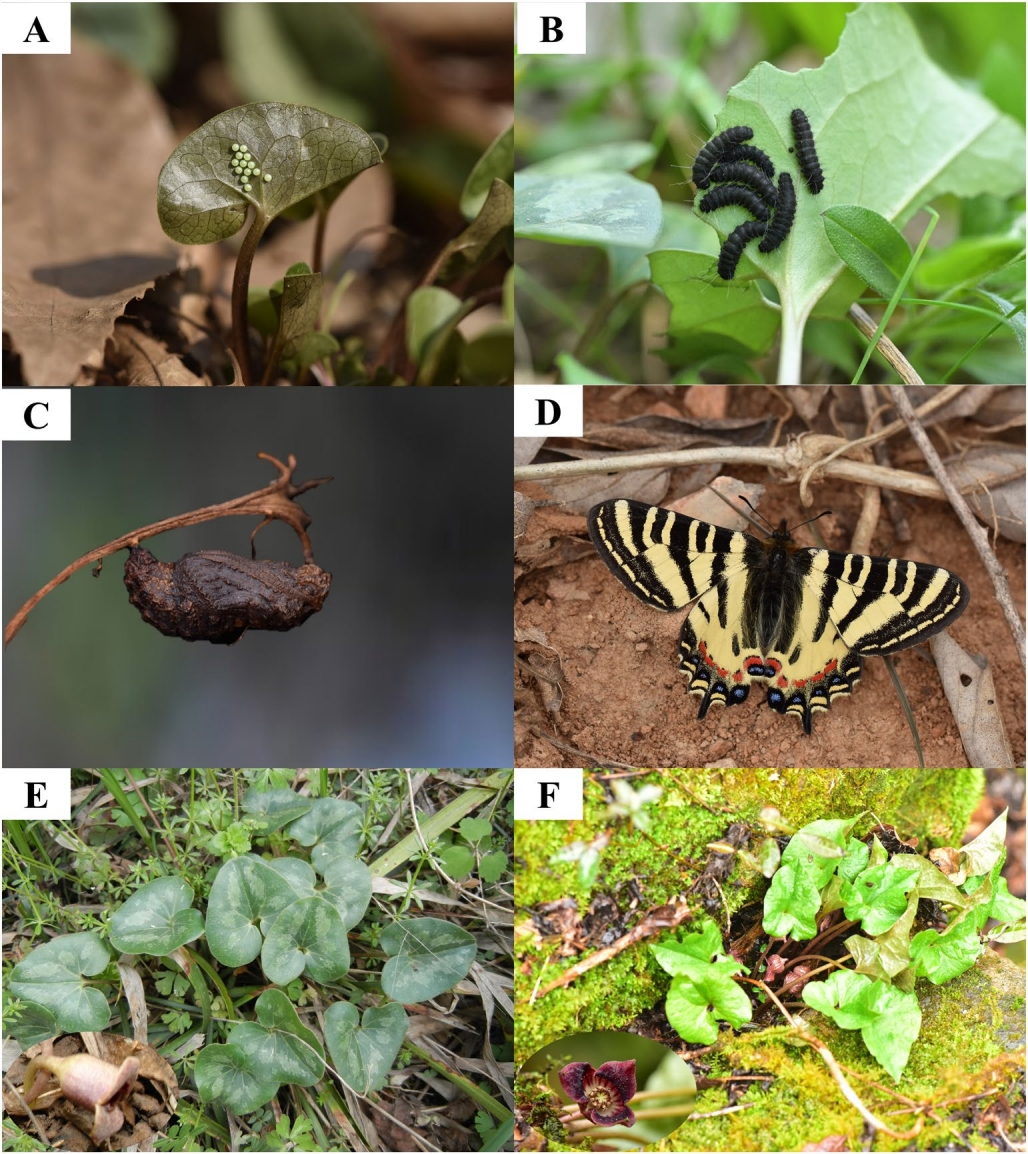
*Luehdorfia chinensis chinensis* and its two host plants. A: *L. c. chinensis* eggs; B: *L. c. chinensis* larvae; C: *L. c. chinensis* pupa; D: *L. c. chinensis* adult (male); E: *Asarum forbesii*; F: *A. sieboldii* . The enlarged flower of each host plant is depicted at the bottom left. Gao Fan took photos A–C, Bao Haoran took photo D, and Zhang Guangfu took photos E and F.

*Luehdorfia chinensis* is an endemic butterfly species in China (family Papilionidae, order Lepidoptera). It comprises two subspecies: *L. c. chinensis* (nominate subspecies) and *L. c. huashanensis*. This butterfly undergoes complete metamorphosis with four distinct stages: egg, larva, pupa, and adult. Typically, *L. c. chinensis* lays eggs from mid‐March to early April (Figure 1A); its larvae develop from April to May, passing through five instars (Figure 1B); its pupal stage extends from May of the current year to March of the following year, including two diapauses spanning both summer and winter (Figure 1C); and the adult stage, from March to April of the following year (Figure 1D), lasts for approximately 2 weeks (Xu et al. 1998). Both adult males and females have yellow wings with black markings and colorful, tiger‐like stripes, making them highly attractive (Figure 1A). The larvae are host‐specific, feeding exclusively on the leaves of two Aristolochiaceae species: *Asarum forbesii* Maxim. (Figure 1E) and *A. sieboldii* Miq. (Figure 1F) (Wu 2001; Wang et al. 2010; Zhang et al. 2022). Each of these plants has a solitary flower in the leaf axil, with dark purple perianth (Figure 1E,F), and produces few seeds in its capsule (Song et al. 2015). Consequently, *L. chinensis* has a highly specialized life history strategy and ecological niche. In the field, it produces few generations per year with low rates of pupation and eclosion (Dong et al. 2014). Although butterflies are often celebrated as flagship species for invertebrate conservation, their striking appearance and exceptional ornamental value have made them prime targets for illegal collectors in the global wildlife trade, with Papilionidae taxa commanding particularly high demand (Wang et al. 2023). We therefore assume that *L. chinensis* is subject to analogous illicit‐collection pressures. Indeed, historically overharvesting, combined with this species' intrinsically low reproductive rate and narrow ecological niche, has rendered it at an acute risk of extinction (Yuan et al. 1998). As a result, it has been listed under Category II of China's List of Wildlife under Special State Protection since 2021 (Zhang et al. 2022), and under IUCN Red List criteria, it is classified as Data Deficient (DD) (https://www.iucnredlist.org/species/12405/122604025).

At present, many studies on *L. chinensis* have focused on its population dynamics, genetic diversity, and artificial breeding (Dong et al. 2014), but its geographical distribution remains poorly understood. Hu et al. (1992) reported its occurrences in Nanjing (Jiangsu) and Hangzhou (Zhejiang), eastern China. According to *the Monograph of Chinese Butterflies*, it is distributed in several provinces of Henan, Hubei, Jiangsu, Shaanxi, and Zhejiang (Chou 1994). Tan et al. (2011) reported that it was discovered at Wuyunjie National Nature Reserve in Hunan Province, central China. Furthermore, Xiang et al. (2020) suggested that it was scattered in certain parts of the Qinling Mountains and the Middle–Lower Yangtze River in China. More recently, there were two populations identified at Taohongling National Nature Reserve in Jiangxi Province, eastern China (Chen et al. 2022). Thus, the actual distribution of this species in China remains unclear.

Species Distribution Models (SDMs) use species occurrence records and environmental variables to predict spatiotemporal distribution patterns through machine learning algorithms, particularly in identifying potential geographic distributions of species. Biomod2, as an ensemble model platform, provides 10 species distribution models—including Artificial Neural Networks (ANN), Flexible Discriminant Analysis (FDA), Generalized Additive Models (GAM), etc. (Garcia et al. 2012). By building an ensemble model composed of multiple individual models, the reliability of model predictions can be significantly enhanced (Cai and Zhang 2024). Liu, Zhang, and Zong (2024) employed Biomod2 to predict climate change effects on the endangered butterfly *Teinopalpus aureus* and found that its range would contract in the future climate. Jinga et al. (2021) pointed out that uneven sampling effort for a species with different subspecies may result in SDMs' inaccuracy at the species level because of data‐rich subspecies dominating and poorly sampled taxa misrepresenting. For *L. chinensis* , the known range of the nominate subspecies is much wider than the other subspecies, which is only found in Huashan, Taibai Mountain in Shaanxi Province, and Shirenshan in Henan Province, China (Niu et al. 1995). Therefore, we select the nominate subspecies of this butterfly for the distribution prediction, rather than the *huashanensis* subspecies.

Here, we used *L. c. chinensis* and its two host plants to investigate spatiotemporal distribution patterns under climate change. We built ensemble models using the Biomod2 platform for each species and addressed three key questions: (1) What are the key environmental factors influencing the distributions of *L. c. chinensis* and its host plants? (2) What are their potential suitable habitats under current and future climate scenarios? (3) How do niche overlaps between species pairs (i.e., *L. c. chinensis* vs. *A. forbesii* and *L. c. chinensis* vs. *A. sieboldii* ) change under current and future climates, to reveal the impacts of climate change on host choice? The objective of this study is to determine the potential geographic distribution and key determinants for the endangered butterfly species, which contributes to its conservation management in China. Moreover, it may provide insights into the response mechanism of specialist insects and their host plants under climate change.

## 2 Materials and Methods

### 2.1 Species Occurrence Records

**FIGURE 2:**
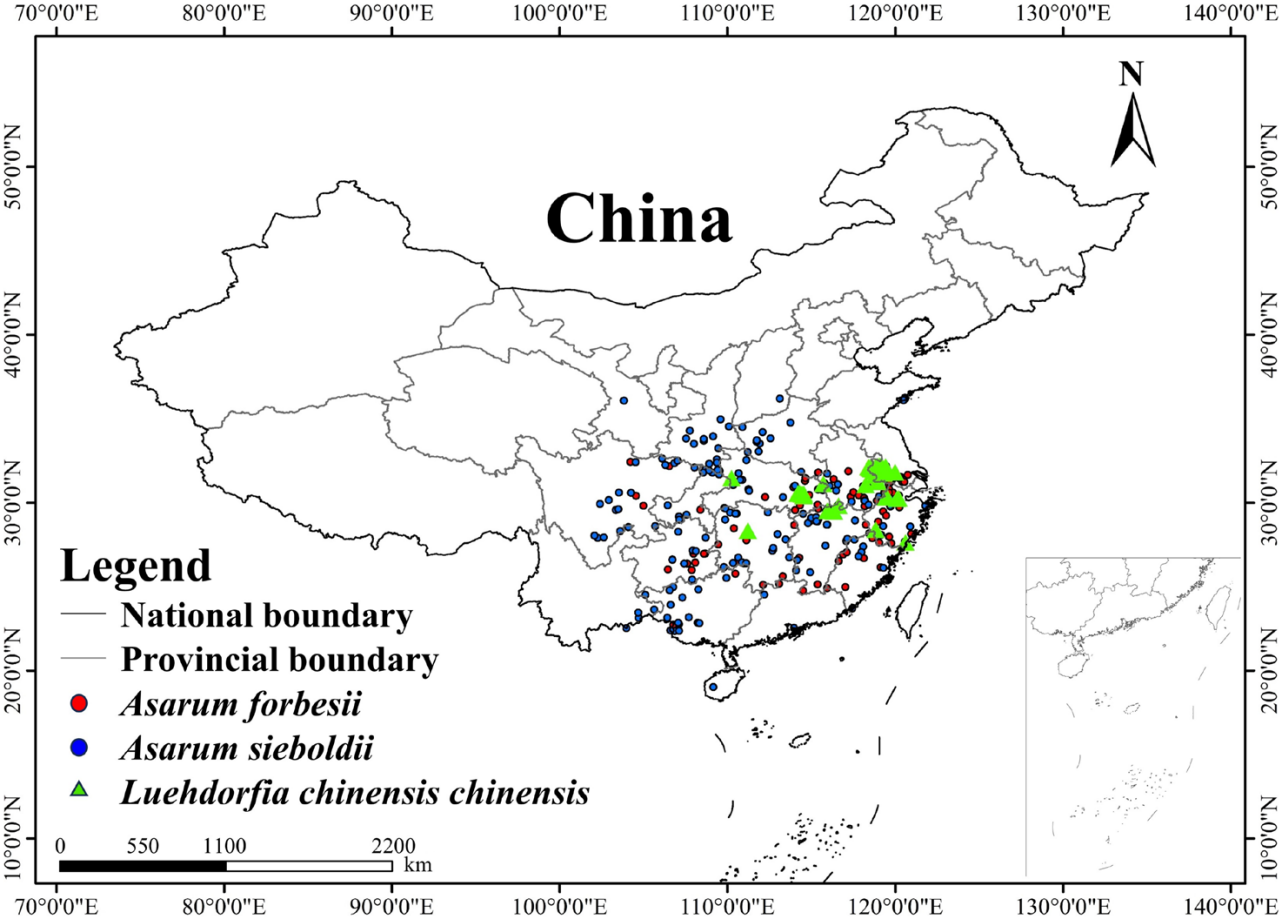
Occurrence records of the endangered butterfly *Luehdorfia chinensis chinensis* (green triangles) and its two host plants, including *Asarum forbesii* (red circles) and *A. sieboldii* (blue circles) in China.

In this study, the occurrence records for *L. c. chinensis* and its host plants, including *A. forbesii* and *A. sieboldii* , were gathered through field surveys, diverse online databases, and relevant literature. Firstly, we conducted surveys of wild populations in Anhui, Jiangsu, Jiangxi, Zhejiang, and other provinces in eastern China over the past 3 years. Next, we obtained each species' original specimen records with precise latitude and longitude or detailed locality data from the National Specimen Information Infrastructure of China (NSII, http://www.nsii.org.cn) and the Global Biodiversity Information Facility (GBIF, https://www.gbif.org/). Thirdly, we searched for each host plant's scientific name in the Plant Picture Bank of China (PPBC, http://ppbc.iplant.cn) to retrieve its locality information, accompanied by images. We used Google Earth to assign latitude and longitude with two decimal places for records lacking specific coordinates. During this initial screening, we excluded records with indeterminate locations and distribution points potentially representing cultivated plants (e.g., botanical gardens, schools, parks). Additionally, we consulted local floras, reports, and related articles (Yao et al. 1999; Tan et al. 2011; Guo et al. 2016). In total, we collected 241 occurrence points for *L. c. chinensis*, 208 for *A. forbesii* , and 199 for *A. sieboldii* . Subsequently, using the Spatially Sparse Occurrence Data for SDMs tool in SDMtoolbox 2.0, we set the spatial resolution at 1 km to minimize sampling bias and spatial autocorrelation (Brown 2014; Mukherjee and Hossain 2024). After processing, we obtained a final dataset of 94 records for *L. c. chinensis*, 156 for *A. forbesii* , and 162 for *A. sieboldii* (Figure 2 and Table S1).

### 2.2 Environmental Variables

Generally, climate is one of the key factors influencing the distribution of species (Lawlor et al. 2024). Based on our field investigation, these two plants mostly grow in the shady and moist mountainous areas under the secondary forests. Moreover, the subspecies butterfly and its food plant *Asarum forbesii* usually occur in the low mountainous areas of eastern and southwestern China, in which there are relatively more human activities on most occasions (Yuan et al. 1998). Thus, three categories of environmental variables were employed in this study. Firstly, we downloaded elevation and aspect data from WorldClim (https://www.worldclim.org/, last accessed on 15 February 2025) and extracted slope data from a Digital Elevation Model (DEM) (http://www.tuxingis.com, last accessed on 15 February 2025). Secondly, we obtained 19 bioclimatic variables from WorldClim, including current and future climate data (i.e., 2041–2060, 2061–2080, and 2081–2100). The current bioclimatic data were downloaded from WorldClim version 2.1 at a resolution of 30 arc‐seconds (~1 km), while future climate projections were derived from the Beijing Climate Center Climate System Model version 2 with Medium Resolution (BCC‐CSM2‐MR) global climate model (CMIP6), which is particularly suitable for Asia and, more specifically, for China (Zhou et al. 2023). We selected three Shared Socioeconomic Pathways (SSPs) (i.e., SSP1‐2.6, SSP2‐4.5, and SSP5‐8.5), representing optimistic, moderate, and pessimistic pathways, respectively (IPCC 2023). Finally, we extracted anthropogenic variables from the global Human Influence (HI) dataset provided by NASA Socioeconomic Data and Applications Center (SEDAC) (https://sedac.ciesin.columbia.edu, last accessed on 8 June 2025), which integrates nine layers including population density, built‐up areas, night‐time lights, land use/cover, and human access pathways (roads, railways, coastlines, and navigable rivers) (Liao et al. 2025). Initially, we considered 23 environmental variables (climatic, topographic, and anthropogenic). To avoid overfitting, we used the “Remove‐Highly‐Correlated‐Variables” tool in SDMtoolbox 2.0, with a maximum correlation threshold of 0.8 (Figure S1). Variables with an absolute Pearson correlation above 0.8 were considered highly correlated; among each correlated pair, the variable with the higher contribution was retained. Ultimately, we selected 10 variables for *L. c. chinensis* (i.e., seven climatic, two topographic, one anthropogenic), 8 variables for *A. forbesii* (i.e., five climatic, two topographic, one anthropogenic), and 9 variables for *A. sieboldii* (i.e., five climatic, three topographic, one anthropogenic) (Figure S1).

### 2.3 Modeling Process

We employed the Biomod2 package (version 3.5.1) to model the current distributions of *L. c. chinensis*, *A. sieboldii* , and *A. forbesii* . Firstly, we ran each of the 10 individual models available on the Biomod2 platform. We then calculated AUC (area under the curve) and TSS (true skill statistic) values for each model. Models with AUC > 0.8 and TSS > 0.7 were selected to create an ensemble model (Cai and Zhang 2024; Hu et al. 2025). We also removed a model with AUC and TSS values of 1. During modeling, 1000 pseudo‐presence points were randomly generated using R 4.4.1, with 75% of the occurrence points used for training and the remaining 25% for validation. This procedure was repeated 10 times, and the mean prediction was taken as the final model output. The AUC and TSS values were calculated for each model (Rathore and Sharma 2023). Models with an AUC greater than 0.9 were classified as excellent, 0.8–0.9 as good, 0.7–0.8 as fair, and 0.6–0.7 as poor (Phillips and Dudík 2008). TSS ranges from −1 to 1, with models classified as excellent (TSS > 0.8), good (0.6–0.8), fair (0.4–0.6), poor (0.2–0.4), and failed (TSS < 0.2) (Allouche et al. 2006).

### 2.4 Geospatial Data Analysis

To visualize potential distribution changes under different climate scenarios, we used ArcMap 10.8 to map the ensemble model results. Based on the “maximum sum of sensitivity and specificity” (Liu et al. 2013), the maxSSS threshold values were 0.2999 for *L. c. chinensis*, 0.2908 for *A. forbesii* , and 0.2713 for *A. sieboldii* . These thresholds allowed us to categorize habitat suitability. For *L. c. chinensis*, suitability was classified as unsuitable (0.00–0.10), low (0.10–0.40), medium (0.40–0.70), and high (0.70–1.00). For *A. forbesii* . unsuitable (0.00–0.13), low suitable (0.13–0.42), medium suitable (0.42–0.71), and highly suitable (0.71–1.00). For *A. sieboldii* , unsuitable (0.00–0.19), low suitable (0.19–0.46), medium suitable (0.46–0.73), and highly suitable (0.73–1.00). For the butterfly, total suitable habitat was defined as the sum of low, medium, and high suitability (Liu, Zhang, and Zong 2024; Liu, Deng, et al. 2024); for each of the plants, it was the sum of medium and high suitability (Wang et al. 2024).

Based on the ArcGIS v10.8 platform, we conducted binary reclassification of potentially suitable distribution data for each host by designating a value of 1 to suitable area and 0 to unsuitable area (Zhang et al. 2019). We then merged the suitable habitats of the two hosts using the Spatial Analyst toolbox. The distribution of *L. c. chinensis* was overlaid with this combined host habitat layer. After standardizing the coordinate system and resolution, raster algebra was applied to extract overlapping regions between the butterfly's distribution and the joint host habitats (Wang et al. 2022); to identify areas suitable for both the butterfly and its hosts. In addition, we used the SDM Toolbox v2.5 to calculate centroid shifts and assess the effects of climate change on species distributions.

### 2.5 Niche Overlap Metrics

The niche overlap index quantifies the similarity between two species in terms of resource use and habitat preferences (Lu et al. 2022). We used two indices, *D* and *I*, to measure niche overlap for the species pairs (*L. c. chinensis* vs. *A. forbesii* , and *L. c. chinensis* vs. *A. sieboldii* ) using ENMTools v1.3.1. The formula for calculating the *D* index is as follows (Warren et al. 2008):

$$D\left(p_{X,}p_{Y}\right)=1-\frac{1}{2}\sum_{i}|p_{X,i}-p_{Y,i}|$$

The suitability values for species *X* and *Y* in a cell where Schoener's *D* ranges from 0 (no overlap) to 1 (complete overlap) represent the similarity of species distributions by comparing the values of each grid cell in the two distributions (Broennimann et al. 2012). *D* values are classified as: Extremely high overlap (0.80–1.00); High overlap (0.60–0.80); Moderate overlap (0.40–0.60); Low overlap (0.20–0.40); and No or very low overlap (0.00–0.20) (Rödder and Engler 2011; Zhou et al. 2023). The *I* index is calculated as:

$$I\left(p_{X,}p_{Y}\right)=\frac{1}{2}\sqrt{\sum_{i}{\left(\sqrt{p_{X,i}}-\sqrt{p_{Y,i}}\right)}^{2}}$$

The *I* index ranges from 0 to 1, where 0 indicates no overlap and 1 indicates complete overlap of environmental requirements (Schoener 1970; Warren et al. 2008).

In addition, the statistical significance of ecological niche overlap differences across climate scenarios, as expressed by Schoener's *D* and Hellinger's *I*, was assessed using a one‐sample *t*‐test; statistical analyses were performed using SPSS 20 (Xue 2024).

## 3 Results

### 3.1 Model Performance

We used Biomod2 to build 10 models for *L. c. chinensis*, *A. forbesii* , and *A. sieboldii* . The final dataset included 94 records and 10 variables for *L. c. chinensis*, 156 records and 8 variables for *A. forbesii* , and 162 records and 9 variables for *A. sieboldii* . Model accuracy of both individual and combined models for each species was evaluated using AUC and TSS (Table 1). For *L. c. chinensis* and *A. sieboldii* , the ANN and SRE models had AUC and TSS values of less than 0.8 and 0.7, respectively, and for *A. forbesii*, only the SRE model fell below those thresholds. These models were excluded from further analysis. The RF model was also excluded due to outlier AUC and TSS values.

**TABLE 1:** Mean values of area under the curve (AUC) and true skill statistic (TSS) for each modeling algorithm and integrated model (bold) for *Luehdorfia chinensis chinensis* and its two host plants under the current climate scenario.

Model	Butterfly: *L. c. chinensis*		Host: *A. forbesii*		Host: *A. sieboldii*	
	AUC	TSS	AUC	TSS	AUC	TSS
RF	1.000	1.000	1.000	1.000	1.000	1.000
SRE	0.767	0.533	0.762	0.523	0.710	0.420
ANN	0.725	0.449	0.890	0.780	0.731	0.456
CTA	0.957	0.899	0.931	0.847	0.896	0.767
FDA	0.970	0.910	0.956	0.837	0.903	0.740
GAM	0.984	0.934	0.959	0.835	0.904	0.747
GBM	0.996	0.956	0.983	0.893	0.954	0.801
GLM	0.978	0.939	0.975	0.869	0.937	0.767
MARS	0.993	0.940	0.978	0.868	0.949	0.802
MAXENT	0.928	0.856	0.964	0.865	0.952	0.772
**Ensemble model**	0.997	0.958	0.978	0.858	0.952	0.783

Conversely, the remaining seven models for *L. c. chinensis* and *A. sieboldii* , and the remaining eight for *A. forbesii* , exhibited AUC > 0.8 and TSS > 0.7, indicating high predictive accuracy. For each species, these models were used to construct an ensemble model. The ensemble model for *L. c. chinensis* achieved an AUC of 0.997 and TSS of 0.958, outperforming individual models and indicating reliability. Similarly, the ensemble models for each host plant outperformed their models, with *A. forbesii* achieving AUC and TSS values of 0.978 and 0.858, respectively, and *A. sieboldii* attaining values of 0.952 and 0.783, respectively.

### 3.2 Contribution of Environmental Variables

The percentage contribution of environmental variables indicated their importance in species distribution modeling (Bradie and Leung 2017). For *L. c. chinensis*, bioclimatic variables accounted for 87.6% of the contribution, and non‐climatic variables contributed 12.4% (Table 2), suggesting climate played a more significant role. For the host *A. forbesii* , bioclimatic variables contributed 75.1% and non‐climatic 24.9%. Similarly, for the host *A. sieboldii* , bioclimatic variables contributed 79.3% and non‐climatic variables 20.7%. Thus, bioclimatic factors appeared to play a greater role than non‐climatic factors in shaping the distribution of these host plants.

**TABLE 2:** Four key environmental variables affecting the habitat distribution of *Luehdorfia chinensis chinensis* and its two host plants.

Species	No.	Variable	Percent contribution (%)
Butterfly: *L. c. chinensis*	1	Bio 17	56.4
	2	Bio 4	16.8
	3	HI	8.2
	4	Bio 9	7.2
Host: *A. forbesii*	1	Bio 14	65.6
	2	HI	20.7
	3	Bio 4	5.0
	4	Slope	3.3
Host: *A. sieboldii*	1	Bio 12	38.8
	2	Bio 6	28.8
	3	HI	13.7
	4	Bio 4	4.7

For *L. c. chinensis*, the top four variables were precipitation of the driest quarter (Bio17, 56.4%), temperature seasonality (Bio4, 16.8%), human influence (HI, 8.2%), and mean temperature of the driest quarter (Bio9, 7.4%), together accounting for 88.8%. Therefore, these four were identified as key factors, with Bio17 being the most important. For *A. forbesii* , the top four variables were the precipitation of the driest month (Bio14, 65.6%), human influence (HI, 20.7%), temperature seasonality (Bio4, 5.0%), and slope (3.3%), totaling 94.6%. For *A. sieboldii* , the top four were annual precipitation (Bio12, 38.3%), minimum temperature of the coldest month (Bio6, 28.8%), human influence (HI, 13.7%), and temperature seasonality (Bio4, 4.7%), totaling 85.5%.

### 3.3 Current Potential Suitable Distribution of *L. c. chinensis* and Its Hosts

**FIGURE 3:**
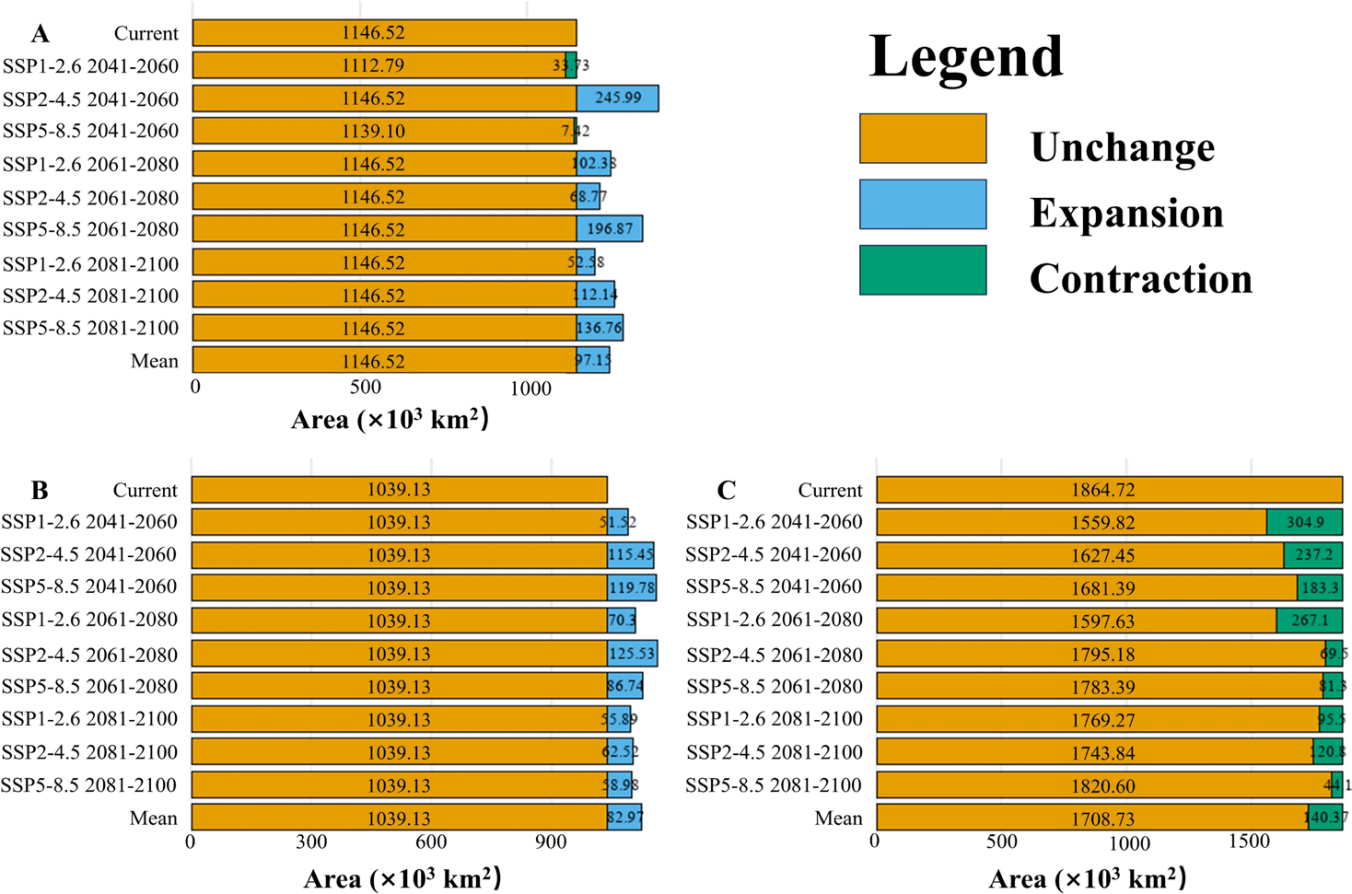
Projected changes in suitable habitat distribution for (A) *Luehdorfia chinensis chinensis*, (B) *Asarum forbesii*, and (C) *A. sieboldii* under future climate scenarios. Mean is the average of the suitable habitat areas of each species under nine future climate scenarios.

**FIGURE 4:**
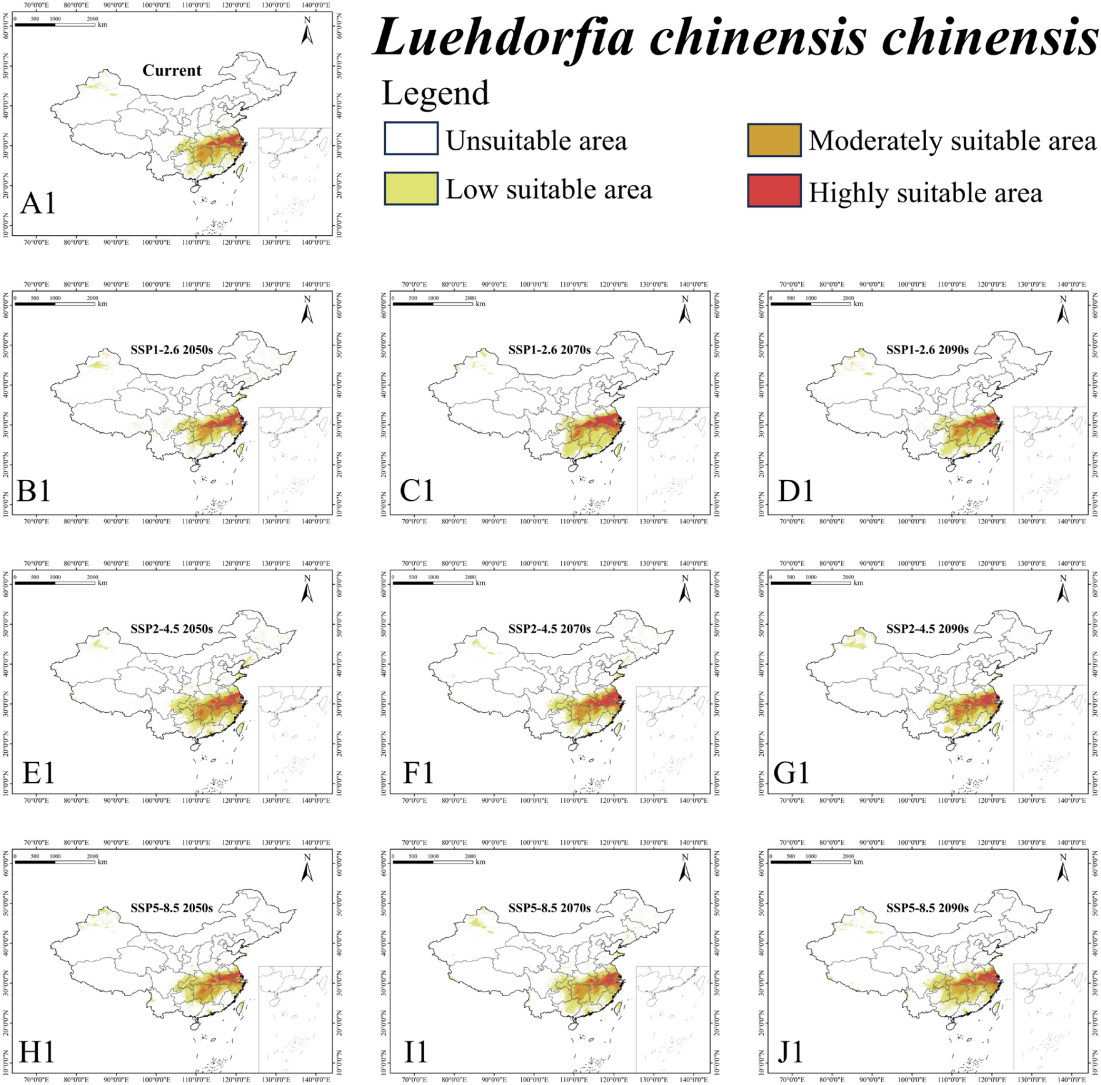
Habitat suitability of *Luehdorfia chinensis chinensis* (A1–J1) in China under different climate scenarios.

**FIGURE 5:**
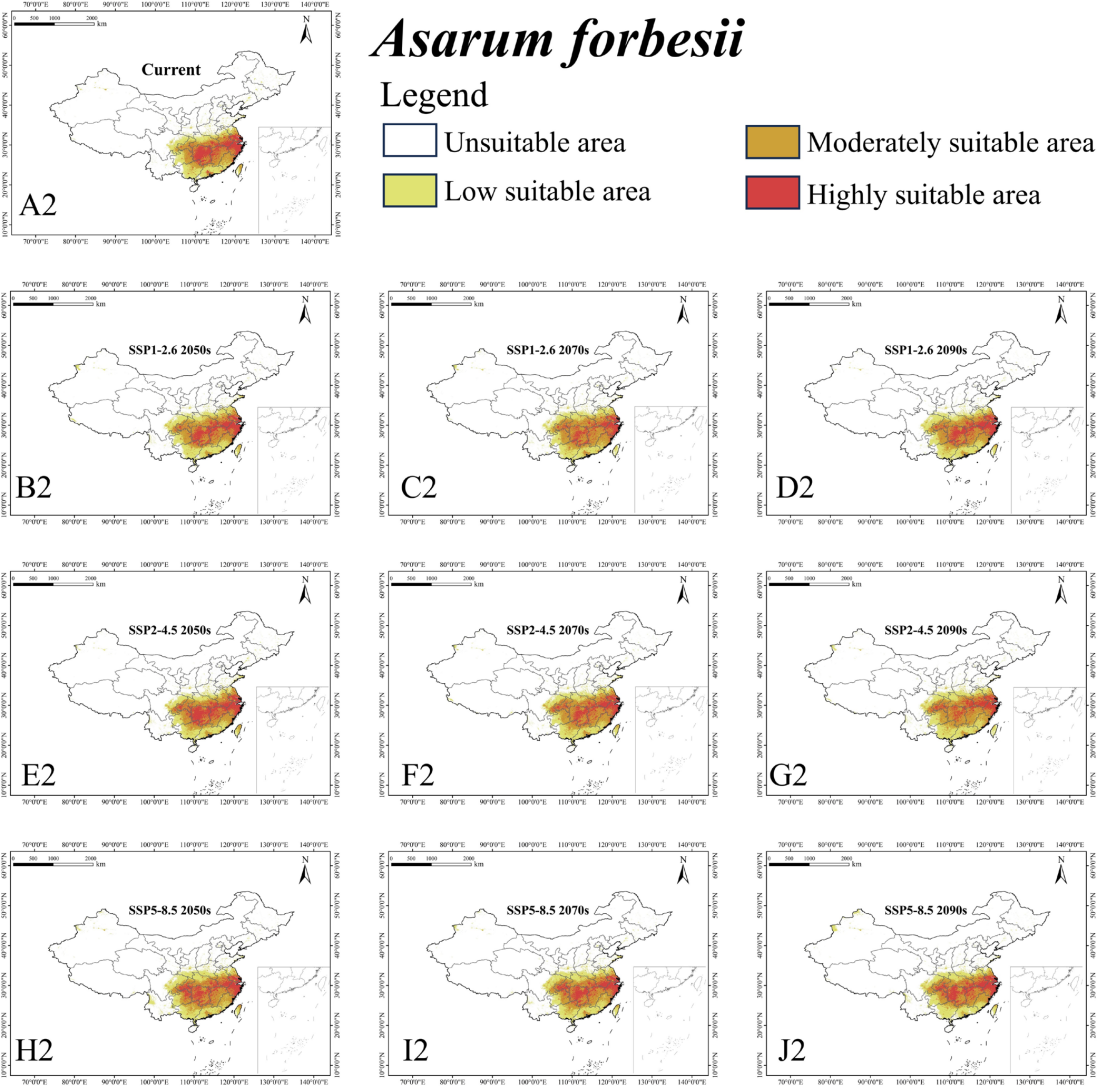
Habitat suitability of *Asarum forbesii* (A2–J2) suitable areas within China under different climate scenarios.

**FIGURE 6:**
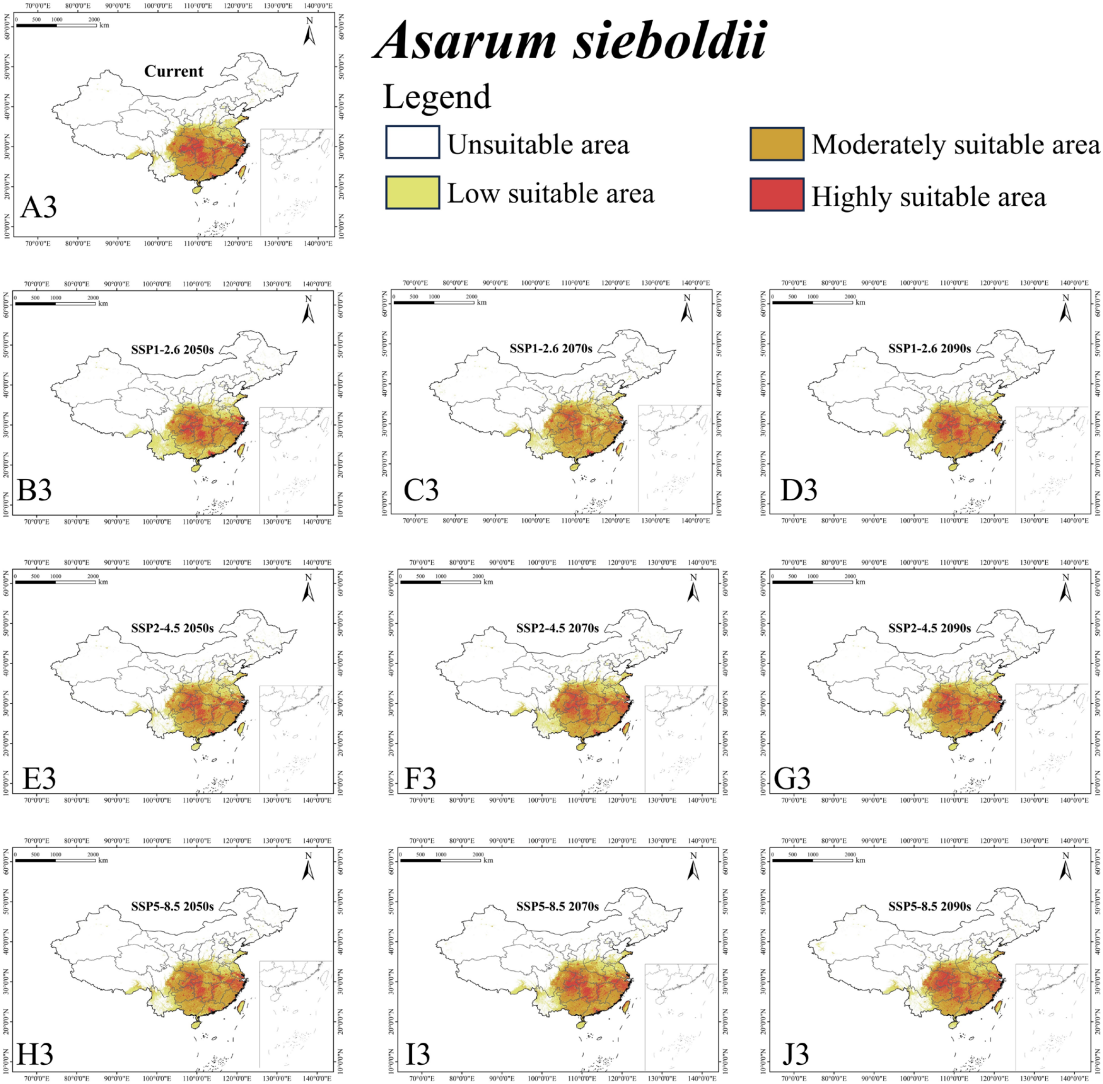
Habitat suitability of *Asarum sieboldii* (A3–J3) in suitable areas within China under different climate scenarios.

**FIGURE 7:**
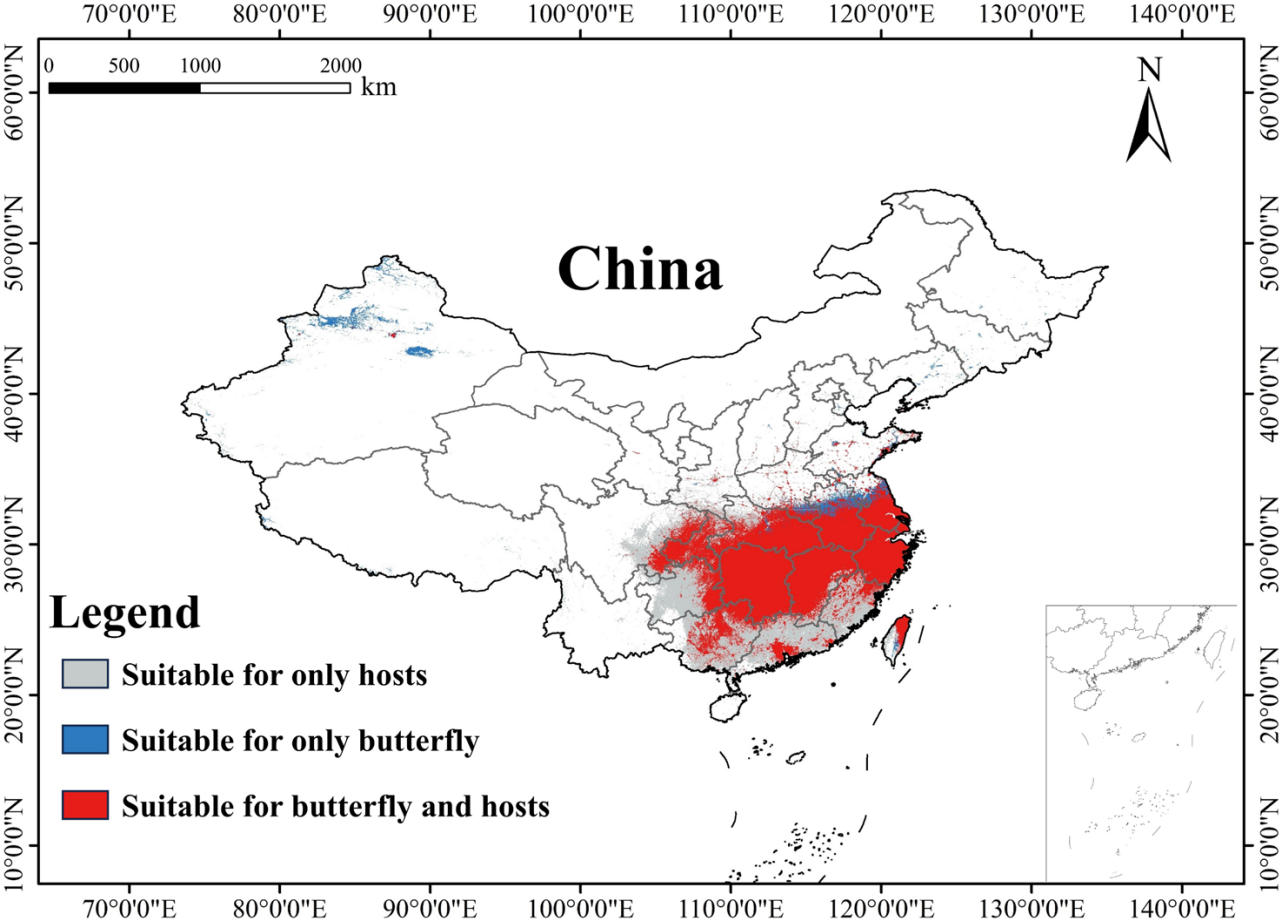
The overlap between *Luehdorfia chinensis chinensis* and its two hosts in a suitable area under the current climate scenario.

The ensemble model indicated that under the current climate, *L. c. chinensis* occupied a suitable area (low, moderately, and highly suitable habitat) of 1,146,520 km^2^, representing 11.95% of China's land area (Figure 3 and Table S2). Its highly suitable area was 122,430 km^2^ (1.28%). The butterfly is mainly found in southeastern China (Anhui, Fujian, Jiangsu, Jiangxi, Zhejiang) and some central regions (Chongqing, southeastern Hubei, and Hunan) (Figure 4A2). For *A. forbesii* , the current suitable area was 1,039,130 km^2^ (10.83% of China), with 444,980 km^2^ (4.64%) highly suitable (Figure 3 and Table S2). This host plant was mainly in southern Anhui, northern Fujian, northeastern Guangxi, eastern Guizhou, Hunan, southern Hubei, northern Jiangsu, Jiangxi, and Zhejiang (Figure 5A3). For *A. sieboldii* , the suitable area was 1,864,700 km^2^ (19.43%), with 521,400 km^2^ (5.43%) highly suitable (Figure 3 and Table S2). This host plant was primarily in southern Anhui, Chongqing, Fujian, Guangdong, Guangxi, eastern Guizhou, southwestern Henan, Hubei, Hunan, southern Jiangsu, Jiangxi, southern Shaanxi, eastern Sichuan, and Zhejiang (Figure 6A1). Therefore, the endangered *L. c. chinensis* had a relatively restricted suitable habitat under the current climate. Its range overlapped with its host plants over 1,020,280 km^2^, which was 89.0% of its suitable habitat (Figure 7).

### 3.4 Future Distribution Shift of *L. c. chinensis* and Its Hosts

For *L. c. chinensis*, the suitable habitat area mostly increased under future scenarios, except under SSP1‐2.62050s or SSP5‐8.52050s. The maximum suitable area (1,392,510 km^2^; 14.51% of China) was under SSP2‐4.52050s and the minimum (1,112,790 km^2^; 11.60%) under SSP1‐2.62050s. The average future suitable area is projected as 1,243,670 km^2^, higher than under the current climate (Figure 3A). For *A. forbesii* , suitable habitat increased under all nine future scenarios. The largest area (1,164,660 km^2^; 12.14%) was under SSP2‐4.52070s, and the smallest (1,090,650 km^2^; 11.36%) under SSP1‐2.62050s. The average future suitable area is 1,122,100 km^2^ (Figure 3B). In contrast, *A. sieboldii* showed decreasing suitable habitat under all scenarios. The largest future area (1,820,600 km^2^; 18.97%) was under SSP5‐8.52090s, and the smallest (1,559,800 km^2^; 16.26%) under SSP1‐2.62050s. The average future suitable area is projected to be 1,724,330 km^2^, lower than currently (Figure 3C).

**FIGURE 8:**
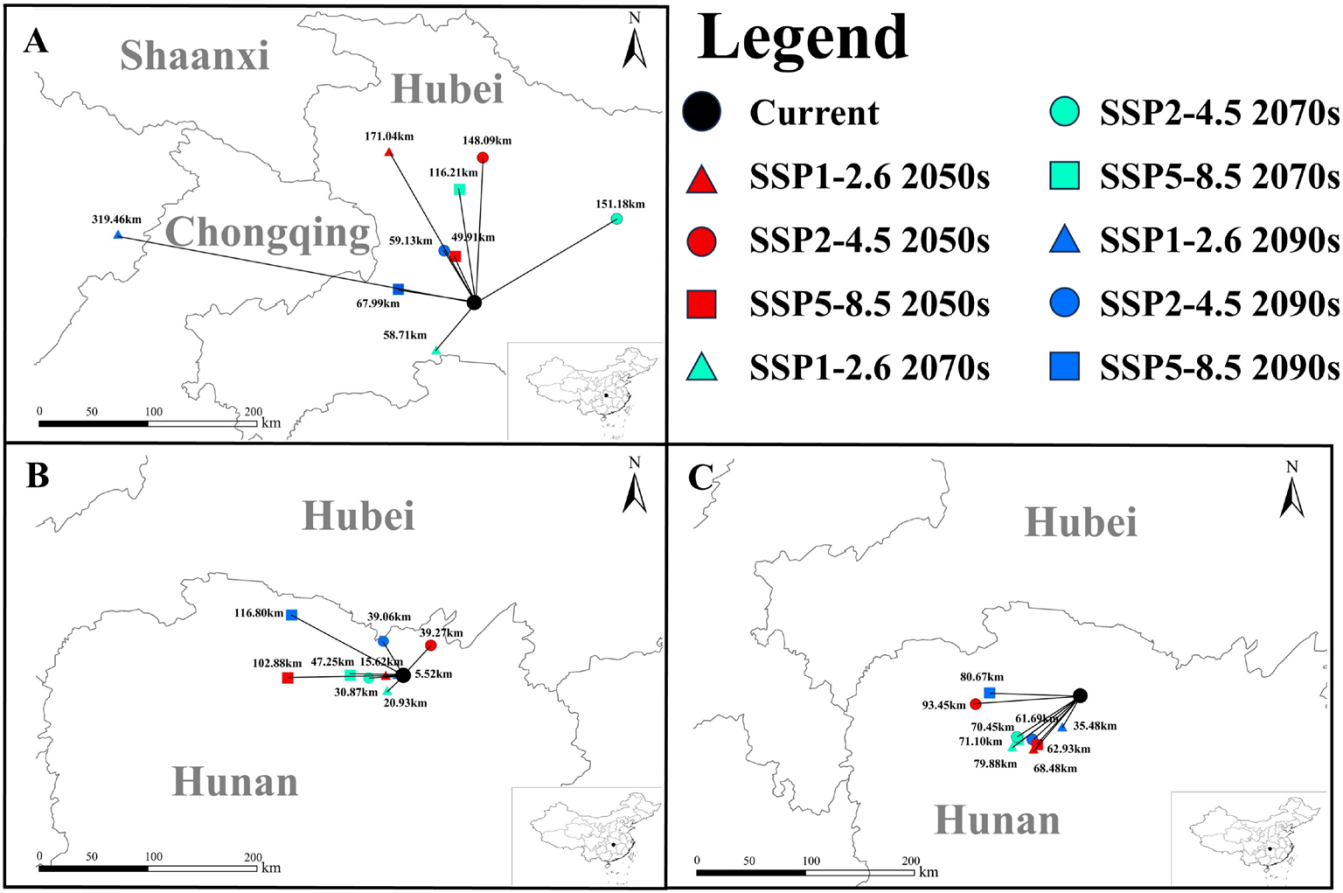
Centroid shift of the *Luehdorfia chinensis chinensis* and its two host plants under different climatic scenarios. A: *L. c. chinensis*; B: *Asarum forbesii*; C: *A. sieboldii* .

The centroid of *L. c. chinensis* shifted northwest under SSP1‐2.62050s, SSP2‐4.52050s, SSP5‐8.52070s, SSP1‐2.62090s, SSP2‐4.52090s, and SSP5‐8.52090s; it moved northeast under SSP2‐4.52070s; and southwest under SSP1‐2.62070s. The average shift distance was 126.86 km, with a maximum of 319.46 km under SSP1‐2.62090s (Figure 8A). The centroid of *A. forbesii* generally shifted southwest under most scenarios, except SSP2‐4.52050s, SSP2‐4.52090s, or SSP5‐8.52090s, with an average shift of 46.47 km and a maximum of 116.80 km under SSP5‐8.52070s (Figure 8B). The centroid of *A. sieboldii* moved predominantly southwest under all future scenarios, with an average shift of 69.35 km and a maximum of 93.45 km under SSP2‐4.52050s (Figure 8C).

### 3.5 Niche Overlap of *L. c. chinensis* and Its Hosts

The current *D* value between *L. c. chinensis* and *A. forbesii* was 0.713, which was classified as high overlap. In contrast, the *D* value between *L. c. chinensis* and *A. sieboldii* was 0.537, which was classified as moderate overlap. Moreover, the current *I* value between *L. c. chinensis* and *A. forbesii* was 0.909, while the *I* value between *L. c. chinensis* and *A. sieboldii* was 0.799 (Table 3). The results of the *I* value are in line with those of the *D* value. Therefore, the former species pair is significantly higher than the latter in terms of niche overlap.

**TABLE 3:** Niche overlap in terms of Schoener's Parameter (*D*) and Hellinger's‐Based Parameter (*I*) of *Luehdorfia chinensis chinensis* and its two host plants under different climatic scenarios.

Climate scenarios		*L. c. chinensis* and *A. forbesii*		*L. c. chinensis* and *A. sieboldii*	
		*D*	*I*	*D*	*I*
Current		0.713	0.909	0.537	0.799
2041–2060	SSP1‐2.6	0.668↓	0.892↓	0.537	0.805↑
	SSP2‐4.5	0.740↑	0.929↑	0.606↑	0.856↑
	SSP5‐8.5	0.689↓	0.903↓	0.551↑	0.817↑
2061–2080	SSP1‐2.6	0.712↓	0.909	0.527↓	0.787↓
	SSP2‐4.5	0.694↓	0.905↓	0.540↑	0.804↑
	SSP5‐8.5	0.734↑	0.921↑	0.591↑	0.844↑
2081–2100	SSP1‐2.6	0.702↓	0.897↓	0.538↑	0.789↓
	SSP2‐4.5	0.695↓	0.902↓	0.537	0.802↑
	SSP5‐8.5	0.745↑	0.922↑	0.580↑	0.829↑
Mean ± SD		0.709 ± 0.026^ns^	0.908 ± 0.012^ns^	0.556 ± 0.028^ns^	0.815 ± 0.024^ns^

*Note:* Mean ± SD refers to the average value of Schoener's *D* and Hellinger's *I* of each species pair under six future climate scenarios. The “ns” indicates no significant difference in niche overlap between current and future situations.

Under nine future climate scenarios, the *D* values for the *L. c. chinensis* and *A. forbesii* species pair showed a decreasing trend, and the *I* value also showed a similar trend. The mean future values (*D* = 0.709, *I* = 0.908) were lower than the current (*D* = 0.713, *I* = 0.909), indicating a slight decrease in niche overlap for this pair. In contrast, under nine future climate scenarios, the *D* value of *L. c. chinensis* and *A. sieboldii* showed an increasing trend. Except for SSP1‐2.62070s or SSP1‐2.62090s, its *I* value also increased, which was similar to its *D* value. For the second pair, the average *D* value in the future (*D* = 0.556) was higher than currently (*D* = 0.537), although its average *I* value in the future (*I* = 0.815) was slightly lower than currently (*I* = 0.799).

## 4 Discussion

### 4.1 Ensemble Model Assessment and Key Environmental Factors

We employed the Biomod2 to build ensemble models for the endangered butterfly *L. c. chinensis* and its two hosts. For each species, the ensemble model showed higher AUC and TSS values than individual models, indicating improved predictive accuracy. This is consistent with Yu and Li (2024), who predicted the distribution of the threatened beetle *Cheirotonus jansoni*. In other words, ensemble models are superior to individual models in terms of prediction reliability when forecasting the potential range of endangered insects.

Model projection (Table 2) indicates that the key factors influencing the distribution of *L. c. chinensis* are bioclimatic rather than topographic. In particular, precipitation of driest quarter (Bio17) contributed nearly 60%, indicating that precipitation in the driest season has a decisive impact on its distribution. It is generally recognized that lepidopteran insects, as key indicators of ecosystem health, are highly sensitive to climate change, and their distributions are predominantly governed by bioclimatic factors (Wilson and Maclean 2011). As a holometabolous insect, *L. c. chinensis* undergoes a pupal stage of over 300 days during the dry season, and it mainly inhabits near‐ground microhabitats (dry branches, tree barks, dead twigs, withered leaves, and crevices) rather than underground or in soil (Yuan et al. 1998). A deficit of precipitation during this stage would reduce ambient humidity in these microhabitats, making adulthood difficult.

Similarly, the most significant factor for the two hosts is Bio14 (driest month precipitation) for *A. forbesii* and Bio12 (annual precipitation) for *A. sieboldii* , with contributions of 65.6% and 38.8%, respectively. Both hosts are perennial herbs of the same genus, *Asarum*, in the family of Aristolochiaceae, and they usually grow to 10 cm (Figure 1E,F), with the main root system distribution in the soil surface. Thus, fluctuations in topsoil moisture directly influence their growth and development (Sun et al. 2018). The anthropogenic factor is identified as a key environmental driver among the top four variables for each species. For example, such a factor contributes over 10% for each host. The possible reason is that *L. c. chinensis* and its hosts are mainly distributed in the humid monsoon lowlands of eastern China (i.e., Jiangsu, Zhejiang, and Anhui provinces), which are economically developed regions with intensive human activities.

In summary, precipitation‐related bioclimatic factors are the primary determinants of habitat suitability for both *L. c. chinensis* and its hosts. Moreover, there is overlap among the top four drivers for the butterfly and its hosts, and a significant overlap in habitat suitability (Figure 6), suggesting that they share similar habitat requirements.

### 4.2 Suitable Range and Future Shift for *L. c. chinensis* and Its Hosts

The current suitable area for *L. c. chinensis* is 1,146,520 km^2^, accounting for approximately 11.95% of China's total land area. This is less than the suitable areas estimated for other threatened swallowtail butterflies, such as *Teinopalpus aureus* (1,389,500 km^2^; 14.47%) (Liu, Zhang, and Zong 2024) and *Troides aeacus* (2,709,600 km^2^; 28.23%) (Liu, Deng, et al. 2024), suggesting that these protected insects have comparatively restricted ranges in China.

Under the current climate, two centers of high suitability for *L. c. chinensis* are identified: (1) the JAZ region, which primarily includes southern Jiangsu, northern Zhejiang, and southeastern Anhui; and (2) the HHJ region, which is mainly concentrated in northern Hunan, southern Hubei, and northern Jiangxi. Under the future climate, the suitable area of *L. c. chinensis* shows a slight increase, with variation among scenarios. Such an increase may be attributed to global warming, causing the butterfly to meet the developmental temperature threshold earlier, to speed up its growth rate and shorten its entire life cycle (Navarro‐Cano et al. 2015).

For the hosts, *A. forbesii* and *A. sieboldii* currently have suitable areas of 1,039,130 km^2^ (10.83%) and 1,864,700 km^2^ (19.43%), respectively. Under future climates, *A. forbesii* increases to the potentially suitable area of 1,122,100 km^2^, whereas *A. sieboldii* slightly decreases to 1,724,330 km^2^, indicating distinct responses to climate change. The centroid of *L. c. chinensis* shifts northwest, mirroring that of *A. forbesii* , whereas *A. sieboldii* shifts southwest, revealing a pronounced divergence between these two congeneric hosts. Both *L. c. chinensis* and *A. forbesii* exhibit range expansions over time, while *A. sieboldii* suffers a pronounced contraction. Overall, *L. c. chinensis* shifts align closely with *A. forbesii* but contrast sharply with *A. sieboldii* .

### 4.3 Niche Overlaps of *L. c. chinensis* and Hosts with Reference to Host Choice

Under future climate conditions, the niche overlap of the first species pair (*L. c. chinensis* vs. *A. forbesii* ) shows a declining trend, whereas the second species pair (*L. c. chinensis* vs. *A. sieboldii* ) exhibits an increasing trend. Field observations and laboratory experiments have confirmed that the two *Asarum* species are the primary food sources for *L. c. chinensis* (Yao et al. 2008; Guo et al. 2016). Therefore, we think that in the coming decades, the host preferences of *L. c. chinensis* will remain largely unchanged because of long‐term coevolution, continuing to specialize in *A. forbesii* and *A. sieboldii* . Consequently, the butterfly *L. c. chinensis* will likely prefer *A. sieboldii* to *A. forbesii* in host choice under climate change, probably resulting in a considerable alteration in its distribution pattern.

Although both hosts occur in China's subtropical mountainous areas, they differ significantly in habitat and elevation. *A. forbesii* is primarily found in the understory of secondary mixed forests at low elevations (< 300 m), whereas *A. sieboldii* occurs in the herb layer of deciduous broadleaved forests at higher elevations (> 1000 m) (Yuan et al. 1998). The observed shift in *L. c. chinensis*'s host preference may imply a migration toward higher altitudes under future climates, similar to movements observed in *Lycaena helle* and other alpine butterflies in Western Europe (Habel et al. 2011; Rödder et al. 2021). Furthermore, niche‐overlap metrics further reveal a projected decline in overlap with *A. forbesii* . Our projections indicate that *L. c. chinensis* faces host mismatch pressure due to climate change. This is like other endangered butterflies, like *Pieris bryoniae* and *Anthocharis euphenoides* (Schweiger et al. 2012). In contrast, the niche overlap with *A. sieboldii* shows a rising trend, providing a climate‐driven adaptive change in host choice for *L. c. chinensis*. Therefore, climate change may drive adaptive shifts in the feeding preferences of *L. c. chinensis* in the future.

### 4.4 Implications for *L. c. chinensis* Conservation

This study is the first to employ an ensemble modeling approach to delineate the climatically suitable distribution of *L. c. chinensis*. We find that suitable habitat spans approximately 1,146,520 km^2^, representing 11.95% of China's total land area, and is primarily distributed in central and southeast China. Precipitation‐related climatic factors are the most critical determinants of the species' distribution. Under future climate change, *L. c. chinensis* may preferentially utilize *A. sieboldii* over *A. forbesii* . These results can inform both in situ and ex situ conservation strategies for this butterfly.

The geographic distribution of *L. c. chinensis* in China has remained unclear, so we recommend conducting additional surveys in projected suitable habitats, especially in the two identified core areas. Xiang et al. (2020) sampled five *L. chinensis* populations from five provinces and conducted the mitochondrial and nuclear gene analysis. They considered that *L*. *c. chinensis* had high genetic diversity, with significant genetic differentiation mainly coming from intrapopulation. Our results show that its current climatically suitable area spans more than 10 provinces, where habitat fragmentation is evident (Figure 3). Therefore, we suggest supplementing genetic sampling in provinces such as Henan and Hubei to fully reflect their genetic diversity and structure in China.

Like other butterflies, *L. c. chinensis* currently occupies a comparatively limited area in China. For this butterfly, its larva needs to undergo five molts, and its pupa needs to experience two diapauses lasting for about 300 days (Zhang et al. 2024). As a result, the species is extremely vulnerable to anthropogenic disturbances. Its two primary hosts, both of which have important medicinal value in China, are currently listed as Near Threatened (*Asarum forbesii*) and Vulnerable ( *A. sieboldii* ), respectively (Qin 2020). Our analysis indicates that human influence (HI) is identified as one of the key drivers shaping the distribution of the three species (Table 2). Therefore, it is critical to mitigate human interference as much as possible within the suitable habitat range of this butterfly. Meanwhile, it is also important to conduct science popularization programs to enhance public conservation awareness. An example in point is the Nanjing Chinese *Luehdorfia* Nature History Museum in Jiangsu Province, eastern China (Zhang and Zhang 2018).

Moreover, in the current climatic context, the distributional overlap between *L*. *c. chinensis* and its host plants provides evidence for the butterfly's host specificity (Figure 6). However, these two host plants respond differently to climate change, which may further influence the suitability of habitats for the butterfly. Therefore, we advocate for climate‐adapted conservation strategies that consider the shifting climate and its effects on both *L. c. chinensis* and its host plants. Notably, since these host plants predominantly occur in secondary forests, we also suggest performing appropriate practices for these forests, which can thereby be maintained at a proper successive stage for the host plants' growth and dispersal.

This study explores the potential distribution of *L. c. chinensis*. It is necessary to investigate the climatically suitable distribution of the *huashanensis* subspecies to improve the conservation and management of the endangered entire species in China.

## 5 Conclusion

We built ensemble models to forecast the potential distribution of the endangered butterfly *L. c. chinensis*, endemic to China, and its two host plants under various climate scenarios. This study is the first to confirm that the butterfly is primarily distributed in central and southeastern China, with a current suitable area of 1,146,520 km^2^; precipitation‐related factors are identified as the primary determinants influencing the distribution of both the butterfly and its two hosts. *L. c. chinensis* is expected to increase moderately in suitable areas under the future climate, and its two hosts exhibit markedly different responses to climate change. Moreover, the butterfly will probably be inclined to alter its larval host plant choice, resulting from global climate change. Our findings highlight that suitable habitats not only for the endangered butterfly but also for its host plants should be taken into account when forming future climate‐adapted conservation strategies.

## Author Contributions

**Ze Lan:** data curation (equal), formal analysis (equal), investigation (equal), writing – original draft (equal). **Guangfu Zhang:** conceptualization (equal), funding acquisition (equal), investigation (equal), supervision (equal), writing – review and editing (equal).

## Conflicts of Interest

The authors declare no conflicts of interest.

## Supporting information


**Figure S1:** Pearson correlation matrix of environmental variables.

**Table S1:** Occurrence records of *Luehdorfia chinensis chinensis*, *Asarum forbesii*, and *A. sieboldii* in China.

**Table S2:** Dynamics of the projected suitable habitats of *Luehdorfia chinensis chinensis* and its two hosts under different climate scenarios.

## Data Availability

The original data are contained within the article and [Link], [Link], [Link].
