# Supplementary figures and images for: Range Shifts of the Endangered Luehdorfia chinensis chinensis (Lepidoptera, Papilionidae) and Its Specific Hosts in China Under Climate Change
Source: Ecol Evol. 2025 Aug 23;15(8):e72057. doi: 10.1002/ece3.72057 (PMC12374071; doi:10.1002/ece3.72057)

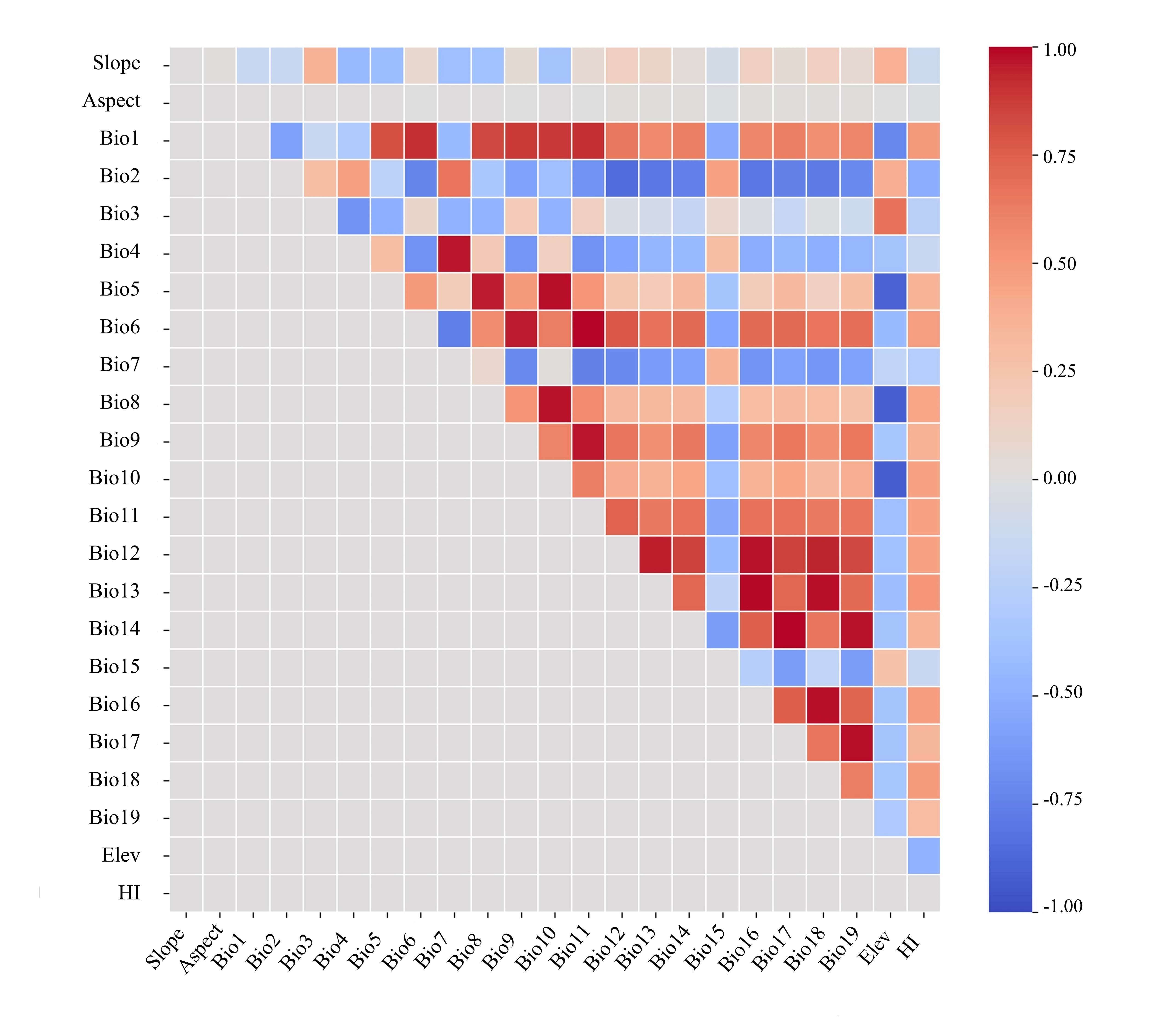

Supplement: Supplementary file 1 — Figure S1: Pearson correlation matrix of environmental variables. [file ECE3-15-e72057-s003.jpg]
