# Supplementary material for: Range Shifts of the Endangered Luehdorfia chinensis chinensis (Lepidoptera, Papilionidae) and Its Specific Hosts in China Under Climate Change
Source: Ecol Evol. 2025 Aug 23;15(8):e72057. doi: 10.1002/ece3.72057 (PMC12374071; doi:10.1002/ece3.72057)
Supplement: Supplementary file 2 — Table S1: Occurrence records of Luehdorfia chinensis chinensis, Asarum forbesii, and A. sieboldii in China. [file ECE3-15-e72057-s001.docx]

**Supplementary Table 1**

Occurrence records of *Luehdorfia chinensis chinensis*, *Asarum forbesii*, and *A. sieboldii* in China.

| **Species** | **Longitude (°)** | **Latitude (°)** |
| --- | --- | --- |
| *Butterfly:Luehdorfia chinensis chinensis* | 119.03 | 32.06 |
| Butterfly*:Luehdorfia chinensis chinensis* | 118.62 | 32.12 |
| Butterfly*:Luehdorfia chinensis chinensis* | 119.02 | 32.07 |
| Butterfly*:Luehdorfia chinensis chinensis* | 118.83 | 32.07 |
| Butterfly*:Luehdorfia chinensis chinensis* | 119.01 | 32.07 |
| Butterfly*:Luehdorfia chinensis chinensis* | 119.02 | 32.06 |
| Butterfly*:Luehdorfia chinensis chinensis* | 118.55 | 32.05 |
| Butterfly*:Luehdorfia chinensis chinensis* | 119.05 | 32.05 |
| Butterfly*:Luehdorfia chinensis chinensis* | 119.08 | 32.13 |
| Butterfly*:Luehdorfia chinensis chinensis* | 118.60 | 32.10 |
| Butterfly*:Luehdorfia chinensis chinensis* | 119.02 | 32.05 |
| Butterfly*:Luehdorfia chinensis chinensis* | 119.18 | 32.02 |
| Butterfly*:Luehdorfia chinensis chinensis* | 118.88 | 31.89 |
| Butterfly*:Luehdorfia chinensis chinensis* | 119.06 | 32.07 |
| Butterfly*:Luehdorfia chinensis chinensis* | 114.31 | 30.59 |
| Butterfly*:Luehdorfia chinensis chinensis* | 119.10 | 32.15 |
| Butterfly*:Luehdorfia chinensis chinensis* | 119.18 | 32.05 |
| Butterfly*:Luehdorfia chinensis chinensis* | 114.20 | 30.56 |
| Butterfly*:Luehdorfia chinensis chinensis* | 119.09 | 32.05 |
| Butterfly*:Luehdorfia chinensis chinensis* | 118.80 | 32.06 |
| Butterfly*:Luehdorfia chinensis chinensis* | 119.06 | 32.06 |
| Butterfly*:Luehdorfia chinensis chinensis* | 114.30 | 30.54 |
| Butterfly*:Luehdorfia chinensis chinensis* | 119.04 | 32.16 |
| Butterfly*:Luehdorfia chinensis chinensis* | 119.03 | 32.18 |
| Butterfly*:Luehdorfia chinensis chinensis* | 119.12 | 32.08 |
| Butterfly*:Luehdorfia chinensis chinensis* | 118.42 | 32.12 |
| Butterfly*:Luehdorfia chinensis chinensis* | 118.24 | 31.07 |
| Butterfly*:Luehdorfia chinensis chinensis* | 119.09 | 32.02 |
| Butterfly*:Luehdorfia chinensis chinensis* | 119.03 | 32.01 |
| Butterfly*:Luehdorfia chinensis chinensis* | 118.85 | 28.40 |
| Butterfly*:Luehdorfia chinensis chinensis* | 118.96 | 32.00 |
| Butterfly*:Luehdorfia chinensis chinensis* | 118.54 | 32.07 |
| Butterfly*:Luehdorfia chinensis chinensis* | 118.94 | 32.01 |
| Butterfly*:Luehdorfia chinensis chinensis* | 120.20 | 30.25 |
| Butterfly*:Luehdorfia chinensis chinensis* | 118.79 | 32.07 |
| Butterfly*:Luehdorfia chinensis chinensis* | 119.43 | 32.16 |
| Butterfly*:Luehdorfia chinensis chinensis* | 119.02 | 32.18 |
| Butterfly*:Luehdorfia chinensis chinensis* | 119.12 | 32.11 |
| Butterfly*:Luehdorfia chinensis chinensis* | 119.18 | 32.18 |
| Butterfly*:Luehdorfia chinensis chinensis* | 119.17 | 32.08 |
| Butterfly*:Luehdorfia chinensis chinensis* | 119.03 | 32.16 |
| Butterfly*:Luehdorfia chinensis chinensis* | 119.09 | 32.17 |
| Butterfly*:Luehdorfia chinensis chinensis* | 114.54 | 30.40 |
| Butterfly*:Luehdorfia chinensis chinensis* | 119.20 | 32.18 |
| Butterfly*:Luehdorfia chinensis chinensis* | 119.01 | 32.11 |
| Butterfly*:Luehdorfia chinensis chinensis* | 119.11 | 32.02 |
| Butterfly*:Luehdorfia chinensis chinensis* | 119.08 | 32.04 |
| Butterfly*:Luehdorfia chinensis chinensis* | 119.03 | 32.15 |
| Butterfly*:Luehdorfia chinensis chinensis* | 119.08 | 32.08 |
| Butterfly*:Luehdorfia chinensis chinensis* | 119.18 | 32.06 |
| Butterfly*:Luehdorfia chinensis chinensis* | 119.02 | 32.08 |
| Butterfly*:Luehdorfia chinensis chinensis* | 119.18 | 32.11 |
| Butterfly*:Luehdorfia chinensis chinensis* | 119.14 | 32.08 |
| Butterfly*:Luehdorfia chinensis chinensis* | 118.58 | 32.01 |
| Butterfly*:Luehdorfia chinensis chinensis* | 118.55 | 32.08 |
| Butterfly*:Luehdorfia chinensis chinensis* | 119.10 | 32.13 |
| Butterfly*:Luehdorfia chinensis chinensis* | 116.63 | 29.82 |
| Butterfly*:Luehdorfia chinensis chinensis* | 119.15 | 32.15 |
| Butterfly*:Luehdorfia chinensis chinensis* | 119.11 | 32.12 |
| Butterfly*:Luehdorfia chinensis chinensis* | 119.12 | 32.18 |
| Butterfly*:Luehdorfia chinensis chinensis* | 118.59 | 32.11 |
| Butterfly*:Luehdorfia chinensis chinensis* | 118.59 | 32.10 |
| Butterfly*:Luehdorfia chinensis chinensis* | 118.93 | 32.18 |
| Butterfly*:Luehdorfia chinensis chinensis* | 118.84 | 32.07 |
| Butterfly*:Luehdorfia chinensis chinensis* | 119.99 | 31.81 |
| Butterfly*:Luehdorfia chinensis chinensis* | 119.31 | 31.78 |
| Butterfly*:Luehdorfia chinensis chinensis* | 118.86 | 31.90 |
| Butterfly*:Luehdorfia chinensis chinensis* | 118.57 | 32.07 |
| Butterfly*:Luehdorfia chinensis chinensis* | 114.44 | 30.51 |
| Butterfly*:Luehdorfia chinensis chinensis* | 118.51 | 32.05 |
| Butterfly*:Luehdorfia chinensis chinensis* | 114.56 | 30.42 |
| Butterfly*:Luehdorfia chinensis chinensis* | 118.96 | 31.27 |
| Butterfly*:Luehdorfia chinensis chinensis* | 119.32 | 31.79 |
| Butterfly*:Luehdorfia chinensis chinensis* | 118.52 | 32.06 |
| Butterfly*:Luehdorfia chinensis chinensis* | 119.31 | 31.75 |
| Butterfly*:Luehdorfia chinensis chinensis* | 119.31 | 31.79 |
| Butterfly*:Luehdorfia chinensis chinensis* | 114.55 | 30.54 |
| Butterfly*:Luehdorfia chinensis chinensis* | 118.78 | 32.06 |
| Butterfly*:Luehdorfia chinensis chinensis* | 118.58 | 32.11 |
| Butterfly*:Luehdorfia chinensis chinensis* | 118.85 | 31.65 |
| Butterfly*:Luehdorfia chinensis chinensis* | 118.80 | 31.62 |
| Butterfly*:Luehdorfia chinensis chinensis* | 110.25 | 31.47 |
| Butterfly*:Luehdorfia chinensis chinensis* | 115.71 | 31.12 |
| Butterfly*:Luehdorfia chinensis chinensis* | 115.97 | 29.52 |
| Butterfly*:Luehdorfia chinensis chinensis* | 119.45 | 30.32 |
| Butterfly*:Luehdorfia chinensis chinensis* | 120.62 | 27.64 |
| Butterfly*:Luehdorfia chinensis chinensis* | 118.85 | 32.07 |
| Butterfly*:Luehdorfia chinensis chinensis* | 118.97 | 31.25 |
| Butterfly*:Luehdorfia chinensis chinensis* | 111.22 | 28.33 |
| Butterfly*:Luehdorfia chinensis chinensis* | 118.74 | 31.90 |
| Butterfly*:Luehdorfia chinensis chinensis* | 118.85 | 32.05 |
| Butterfly*:Luehdorfia chinensis chinensis* | 120.10 | 30.24 |
| Butterfly*:Luehdorfia chinensis chinensis* | 116.35 | 29.48 |
| Butterfly*:Luehdorfia chinensis chinensis* | 119.51 | 30.35 |
| Host*: Asarum forbesii* | 119.99 | 31.81 |
| Host*: Asarum forbesii* | 119.14 | 32.16 |
| Host*: Asarum forbesii* | 119.21 | 32.15 |
| Host*: Asarum forbesii* | 119.01 | 32.07 |
| Host*: Asarum forbesii* | 118.86 | 32.07 |
| Host*: Asarum forbesii* | 119.08 | 32.14 |
| Host*: Asarum forbesii* | 119.01 | 32.05 |
| Host*: Asarum forbesii* | 119.01 | 32.04 |
| Host*: Asarum forbesii* | 120.25 | 29.75 |
| Host*: Asarum forbesii* | 119.01 | 32.15 |
| Host*: Asarum forbesii* | 118.90 | 32.01 |
| Host*: Asarum forbesii* | 118.83 | 32.06 |
| Host*: Asarum forbesii* | 114.27 | 26.72 |
| Host*: Asarum forbesii* | 115.95 | 28.68 |
| Host*: Asarum forbesii* | 115.40 | 30.78 |
| Host*: Asarum forbesii* | 118.25 | 28.68 |
| Host*: Asarum forbesii* | 118.18 | 29.78 |
| Host*: Asarum forbesii* | 119.48 | 31.42 |
| Host*: Asarum forbesii* | 119.17 | 31.95 |
| Host*: Asarum forbesii* | 115.38 | 25.13 |
| Host*: Asarum forbesii* | 115.96 | 28.68 |
| Host*: Asarum forbesii* | 110.33 | 31.05 |
| Host*: Asarum forbesii* | 114.52 | 24.75 |
| Host*: Asarum forbesii* | 119.68 | 30.63 |
| Host*: Asarum forbesii* | 116.33 | 31.40 |
| Host*: Asarum forbesii* | 120.95 | 28.13 |
| Host*: Asarum forbesii* | 114.30 | 30.57 |
| Host*: Asarum forbesii* | 107.10 | 29.16 |
| Host*: Asarum forbesii* | 114.03 | 29.55 |
| Host*: Asarum forbesii* | 119.28 | 29.48 |
| Host*: Asarum forbesii* | 119.72 | 30.23 |
| Host*: Asarum forbesii* | 120.75 | 31.65 |
| Host*: Asarum forbesii* | 119.63 | 27.98 |
| Host*: Asarum forbesii* | 120.57 | 27.67 |
| Host*: Asarum forbesii* | 110.38 | 28.47 |
| Host*: Asarum forbesii* | 104.55 | 30.4 |
| Host*: Asarum forbesii* | 115.67 | 30.75 |
| Host*: Asarum forbesii* | 118.13 | 30.3 |
| Host*: Asarum forbesii* | 119.05 | 27.62 |
| Host*: Asarum forbesii* | 119.45 | 29.22 |
| Host*: Asarum forbesii* | 108.68 | 26.97 |
| Host*: Asarum forbesii* | 118.85 | 31.95 |
| Host*: Asarum forbesii* | 119.03 | 32.06 |
| Host*: Asarum forbesii* | 118.94 | 32.00 |
| Host*: Asarum forbesii* | 119.09 | 32.13 |
| Host*: Asarum forbesii* | 119.21 | 32.13 |
| Host*: Asarum forbesii* | 118.10 | 30.98 |
| Host*: Asarum forbesii* | 118.55 | 32.06 |
| Host*: Asarum forbesii* | 115.41 | 29.62 |
| Host*: Asarum forbesii* | 114.31 | 30.55 |
| Host*: Asarum forbesii* | 120.17 | 30.27 |
| Host*: Asarum forbesii* | 110.85 | 26.43 |
| Host*: Asarum forbesii* | 115.40 | 31.79 |
| Host*: Asarum forbesii* | 119.17 | 31.92 |
| Host*: Asarum forbesii* | 120.32 | 31.53 |
| Host*: Asarum forbesii* | 118.80 | 32.06 |
| Host*: Asarum forbesii* | 118.18 | 29.79 |
| Host*: Asarum forbesii* | 119.82 | 31.38 |
| Host*: Asarum forbesii* | 120.41 | 31.24 |
| Host*: Asarum forbesii* | 115.99 | 29.67 |
| Host*: Asarum forbesii* | 117.35 | 30.35 |
| Host*: Asarum forbesii* | 117.51 | 30.62 |
| Host*: Asarum forbesii* | 117.20 | 31.87 |
| Host*: Asarum forbesii* | 118.17 | 30.11 |
| Host*: Asarum forbesii* | 118.32 | 31.05 |
| Host*: Asarum forbesii* | 119.61 | 31.12 |
| Host*: Asarum forbesii* | 115.41 | 31.80 |
| Host*: Asarum forbesii* | 114.62 | 31.29 |
| Host*: Asarum forbesii* | 114.65 | 31.46 |
| Host*: Asarum forbesii* | 115.38 | 29.88 |
| Host*: Asarum forbesii* | 114.42 | 30.54 |
| Host*: Asarum forbesii* | 114.44 | 30.53 |
| Host*: Asarum forbesii* | 111.26 | 30.80 |
| Host*: Asarum forbesii* | 111.13 | 27.76 |
| Host*: Asarum forbesii* | 110.55 | 29.35 |
| Host*: Asarum forbesii* | 118.86 | 31.90 |
| Host*: Asarum forbesii* | 118.70 | 32.08 |
| Host*: Asarum forbesii* | 118.64 | 32.07 |
| Host*: Asarum forbesii* | 118.81 | 32.08 |
| Host*: Asarum forbesii* | 119.06 | 32.06 |
| Host*: Asarum forbesii* | 120.55 | 31.23 |
| Host*: Asarum forbesii* | 119.70 | 31.21 |
| Host*: Asarum forbesii* | 120.25 | 31.45 |
| Host*: Asarum forbesii* | 119.08 | 32.13 |
| Host*: Asarum forbesii* | 119.30 | 32.06 |
| Host*: Asarum forbesii* | 119.45 | 32.18 |
| Host*: Asarum forbesii* | 114.96 | 28.93 |
| Host*: Asarum forbesii* | 115.88 | 29.51 |
| Host*: Asarum forbesii* | 116.05 | 29.45 |
| Host*: Asarum forbesii* | 115.36 | 28.87 |
| Host*: Asarum forbesii* | 115.40 | 29.00 |
| Host*: Asarum forbesii* | 103.48 | 29.60 |
| Host*: Asarum forbesii* | 119.22 | 30.16 |
| Host*: Asarum forbesii* | 120.22 | 30.43 |
| Host*: Asarum forbesii* | 119.72 | 29.77 |
| Host*: Asarum forbesii* | 119.66 | 29.92 |
| Host*: Asarum forbesii* | 120.12 | 30.22 |
| Host*: Asarum forbesii* | 120.00 | 30.12 |
| Host*: Asarum forbesii* | 119.61 | 30.49 |
| Host*: Asarum forbesii* | 119.81 | 30.51 |
| Host*: Asarum forbesii* | 120.01 | 30.19 |
| Host*: Asarum forbesii* | 120.13 | 30.26 |
| Host*: Asarum forbesii* | 120.16 | 30.24 |
| Host*: Asarum forbesii* | 119.79 | 30.24 |
| Host*: Asarum forbesii* | 119.64 | 30.80 |
| Host*: Asarum forbesii* | 119.68 | 30.53 |
| Host*: Asarum forbesii* | 119.35 | 30.58 |
| Host*: Asarum forbesii* | 119.86 | 30.60 |
| Host*: Asarum forbesii* | 119.07 | 28.07 |
| Host*: Asarum forbesii* | 118.91 | 30.11 |
| Host*: Asarum forbesii* | 119.71 | 30.22 |
| Host*: Asarum forbesii* | 118.99 | 28.87 |
| Host*: Asarum forbesii* | 120.25 | 29.72 |
| Host*: Asarum forbesii* | 119.76 | 27.58 |
| Host*: Asarum forbesii* | 119.63 | 30.60 |
| Host*: Asarum forbesii* | 119.45 | 30.29 |
| Host*: Asarum forbesii* | 104.22 | 32.42 |
| Host*: Asarum forbesii* | 110.48 | 25.77 |
| Host*: Asarum forbesii* | 106.78 | 22.73 |
| Host*: Asarum forbesii* | 108.38 | 29.58 |
| Host*: Asarum forbesii* | 113.07 | 25.17 |
| Host*: Asarum forbesii* | 115.95 | 24.90 |
| Host*: Asarum forbesii* | 118.13 | 26.69 |
| Host*: Asarum forbesii* | 118.64 | 27.88 |
| Host*: Asarum forbesii* | 117.02 | 24.97 |
| Host*: Asarum forbesii* | 116.89 | 26.91 |
| Host*: Asarum forbesii* | 118.45 | 28.17 |
| Host*: Asarum forbesii* | 117.11 | 27.04 |
| Host*: Asarum forbesii* | 119.13 | 26.15 |
| Host*: Asarum forbesii* | 109.45 | 27.51 |
| Host*: Asarum forbesii* | 111.00 | 26.38 |
| Host*: Asarum forbesii* | 110.66 | 26.48 |
| Host*: Asarum forbesii* | 113.30 | 25.61 |
| Host*: Asarum forbesii* | 113.98 | 28.66 |
| Host*: Asarum forbesii* | 112.74 | 27.23 |
| Host*: Asarum forbesii* | 107.87 | 25.98 |
| Host*: Asarum forbesii* | 107.60 | 26.37 |
| Host*: Asarum forbesii* | 106.72 | 26.61 |
| Host*: Asarum forbesii* | 108.60 | 26.96 |
| Host*: Asarum forbesii* | 108.08 | 26.42 |
| Host*: Asarum forbesii* | 107.98 | 26.95 |
| Host*: Asarum forbesii* | 105.00 | 29.81 |
| Host*: Asarum forbesii* | 106.53 | 32.17 |
| Host*: Asarum forbesii* | 106.45 | 26.02 |
| Host*: Asarum forbesii* | 112.12 | 25.10 |
| Host*: Asarum forbesii* | 109.81 | 29.68 |
| Host*: Asarum forbesii* | 116.63 | 26.52 |
| Host*: Asarum forbesii* | 117.83 | 30.43 |
| Host*: Asarum forbesii* | 118.14 | 30.27 |
| Host*: Asarum forbesii* | 119.64 | 27.97 |
| Host*: Asarum forbesii* | 116.61 | 29.86 |
| Host*: Asarum forbesii* | 120.15 | 30.21 |
| Host*: Asarum forbesii* | 114.32 | 29.84 |
| Host*: Asarum forbesii* | 114.87 | 30.45 |
| Host*: Asarum forbesii* | 113.92 | 30.92 |
| Host*: Asarum forbesii* | 112.24 | 30.33 |
| Host*:Asarum sieboldii* | 116.58 | 31.09 |
| Host*:Asarum sieboldii* | 114.35 | 30.55 |
| Host*:Asarum sieboldii* | 114.37 | 30.51 |
| Host*:Asarum sieboldii* | 111.92 | 33.52 |
| Host*:Asarum sieboldii* | 104.53 | 32.41 |
| Host*:Asarum sieboldii* | 109.37 | 32.58 |
| Host*:Asarum sieboldii* | 113.31 | 30.32 |
| Host*:Asarum sieboldii* | 114.28 | 30.60 |
| Host*:Asarum sieboldii* | 111.21 | 33.01 |
| Host*:Asarum sieboldii* | 113.76 | 34.77 |
| Host*:Asarum sieboldii* | 120.58 | 36.25 |
| Host*:Asarum sieboldii* | 110.88 | 31.35 |
| Host*:Asarum sieboldii* | 114.02 | 31.01 |
| Host*:Asarum sieboldii* | 110.75 | 31.35 |
| Host*:Asarum sieboldii* | 112.41 | 29.42 |
| Host*:Asarum sieboldii* | 112.67 | 27.13 |
| Host*:Asarum sieboldii* | 109.87 | 29.65 |
| Host*:Asarum sieboldii* | 110.64 | 26.73 |
| Host*:Asarum sieboldii* | 102.93 | 30.15 |
| Host*:Asarum sieboldii* | 114.17 | 26.58 |
| Host*:Asarum sieboldii* | 119.44 | 30.42 |
| Host*:Asarum sieboldii* | 115.77 | 31.16 |
| Host*:Asarum sieboldii* | 115.78 | 31.17 |
| Host*:Asarum sieboldii* | 118.19 | 30.13 |
| Host*:Asarum sieboldii* | 110.09 | 34.54 |
| Host*:Asarum sieboldii* | 103.83 | 36.07 |
| Host*:Asarum sieboldii* | 109.45 | 33.24 |
| Host*:Asarum sieboldii* | 112.57 | 33.83 |
| Host*:Asarum sieboldii* | 111.74 | 33.62 |
| Host*:Asarum sieboldii* | 114.39 | 31.83 |
| Host*:Asarum sieboldii* | 120.48 | 36.11 |
| Host*:Asarum sieboldii* | 106.83 | 32.35 |
| Host*:Asarum sieboldii* | 102.27 | 27.90 |
| Host*:Asarum sieboldii* | 116.05 | 29.45 |
| Host*:Asarum sieboldii* | 108.23 | 22.84 |
| Host*:Asarum sieboldii* | 108.33 | 22.82 |
| Host*:Asarum sieboldii* | 115.92 | 28.68 |
| Host*:Asarum sieboldii* | 114.52 | 29.33 |
| Host*:Asarum sieboldii* | 114.80 | 28.78 |
| Host*:Asarum sieboldii* | 109.53 | 31.89 |
| Host*:Asarum sieboldii* | 106.11 | 32.63 |
| Host*:Asarum sieboldii* | 106.30 | 32.24 |
| Host*:Asarum sieboldii* | 107.03 | 32.50 |
| Host*:Asarum sieboldii* | 102.08 | 28.04 |
| Host*:Asarum sieboldii* | 109.50 | 31.68 |
| Host*:Asarum sieboldii* | 109.11 | 31.78 |
| Host*:Asarum sieboldii* | 108.67 | 31.95 |
| Host*:Asarum sieboldii* | 108.90 | 31.92 |
| Host*:Asarum sieboldii* | 107.93 | 27.15 |
| Host*:Asarum sieboldii* | 109.54 | 31.90 |
| Host*:Asarum sieboldii* | 110.17 | 29.41 |
| Host*:Asarum sieboldii* | 111.32 | 28.14 |
| Host*:Asarum sieboldii* | 115.69 | 30.74 |
| Host*:Asarum sieboldii* | 116.04 | 30.97 |
| Host*:Asarum sieboldii* | 114.99 | 29.09 |
| Host*:Asarum sieboldii* | 111.29 | 30.70 |
| Host*:Asarum sieboldii* | 110.33 | 26.40 |
| Host*:Asarum sieboldii* | 109.79 | 26.16 |
| Host*:Asarum sieboldii* | 107.89 | 25.42 |
| Host*:Asarum sieboldii* | 106.71 | 26.60 |
| Host*:Asarum sieboldii* | 105.70 | 28.60 |
| Host*:Asarum sieboldii* | 107.07 | 28.26 |
| Host*:Asarum sieboldii* | 120.16 | 30.27 |
| Host*:Asarum sieboldii* | 120.90 | 28.58 |
| Host*:Asarum sieboldii* | 119.15 | 28.08 |
| Host*:Asarum sieboldii* | 119.73 | 30.24 |
| Host*:Asarum sieboldii* | 108.91 | 32.31 |
| Host*:Asarum sieboldii* | 108.59 | 33.68 |
| Host*:Asarum sieboldii* | 107.42 | 26.36 |
| Host*:Asarum sieboldii* | 106.44 | 29.84 |
| Host*:Asarum sieboldii* | 119.30 | 26.11 |
| Host*:Asarum sieboldii* | 109.59 | 34.96 |
| Host*:Asarum sieboldii* | 117.57 | 27.79 |
| Host*:Asarum sieboldii* | 115.84 | 27.43 |
| Host*:Asarum sieboldii* | 117.07 | 27.71 |
| Host*:Asarum sieboldii* | 108.70 | 27.92 |
| Host*:Asarum sieboldii* | 111.75 | 33.72 |
| Host*:Asarum sieboldii* | 109.42 | 31.94 |
| Host*:Asarum sieboldii* | 108.12 | 27.11 |
| Host*:Asarum sieboldii* | 113.47 | 27.81 |
| Host*:Asarum sieboldii* | 112.72 | 27.30 |
| Host*:Asarum sieboldii* | 117.60 | 28.94 |
| Host*:Asarum sieboldii* | 103.35 | 29.56 |
| Host*:Asarum sieboldii* | 110.48 | 29.35 |
| Host*:Asarum sieboldii* | 112.99 | 28.12 |
| Host*:Asarum sieboldii* | 113.68 | 26.74 |
| Host*:Asarum sieboldii* | 106.86 | 22.35 |
| Host*:Asarum sieboldii* | 107.11 | 22.76 |
| Host*:Asarum sieboldii* | 115.95 | 29.55 |
| Host*:Asarum sieboldii* | 109.15 | 19.01 |
| Host*:Asarum sieboldii* | 104.09 | 28.54 |
| Host*:Asarum sieboldii* | 119.46 | 30.34 |
| Host*:Asarum sieboldii* | 102.63 | 27.93 |
| Host*:Asarum sieboldii* | 118.09 | 27.39 |
| Host*:Asarum sieboldii* | 106.42 | 23.14 |
| Host*:Asarum sieboldii* | 117.99 | 27.05 |
| Host*:Asarum sieboldii* | 107.11 | 29.16 |
| Host*:Asarum sieboldii* | 116.48 | 30.72 |
| Host*:Asarum sieboldii* | 107.12 | 28.99 |
| Host*:Asarum sieboldii* | 103.53 | 30.59 |
| Host*:Asarum sieboldii* | 108.26 | 24.83 |
| Host*:Asarum sieboldii* | 108.55 | 31.78 |
| Host*:Asarum sieboldii* | 104.71 | 23.13 |
| Host*:Asarum sieboldii* | 104.68 | 23.44 |
| Host*:Asarum sieboldii* | 110.86 | 26.44 |
| Host*:Asarum sieboldii* | 111.11 | 26.39 |
| Host*:Asarum sieboldii* | 113.96 | 22.55 |
| Host*:Asarum sieboldii* | 103.49 | 29.61 |
| Host*:Asarum sieboldii* | 112.19 | 24.52 |
| Host*:Asarum sieboldii* | 102.40 | 29.92 |
| Host*:Asarum sieboldii* | 103.97 | 22.52 |
| Host*:Asarum sieboldii* | 114.09 | 25.96 |
| Host*:Asarum sieboldii* | 110.67 | 31.75 |
| Host*:Asarum sieboldii* | 105.64 | 23.63 |
| Host*:Asarum sieboldii* | 109.86 | 28.99 |
| Host*:Asarum sieboldii* | 109.93 | 25.51 |
| Host*:Asarum sieboldii* | 115.29 | 29.13 |
| Host*:Asarum sieboldii* | 107.56 | 29.27 |
| Host*:Asarum sieboldii* | 117.44 | 23.69 |
| Host*:Asarum sieboldii* | 106.62 | 23.91 |
| Host*:Asarum sieboldii* | 117.82 | 26.8 |
| Host*:Asarum sieboldii* | 106.76 | 24.97 |
| Host*:Asarum sieboldii* | 110.35 | 31.05 |
| Host*:Asarum sieboldii* | 105.10 | 24.50 |
| Host*:Asarum sieboldii* | 116.09 | 30.99 |
| Host*:Asarum sieboldii* | 107.06 | 22.84 |
| Host*:Asarum sieboldii* | 106.61 | 22.39 |
| Host*:Asarum sieboldii* | 106.56 | 24.78 |
| Host*:Asarum sieboldii* | 107.32 | 24.35 |
| Host*:Asarum sieboldii* | 107.70 | 23.17 |
| Host*:Asarum sieboldii* | 107.11 | 22.36 |
| Host*:Asarum sieboldii* | 103.60 | 28.30 |
| Host*:Asarum sieboldii* | 120.60 | 36.20 |
| Host*:Asarum sieboldii* | 110.90 | 34.50 |
| Host*:Asarum sieboldii* | 108.00 | 33.50 |
| Host*:Asarum sieboldii* | 115.40 | 30.80 |
| Host*:Asarum sieboldii* | 112.10 | 34.20 |
| Host*:Asarum sieboldii* | 120.50 | 36.10 |
| Host*:Asarum sieboldii* | 118.19 | 30.10 |
| Host*:Asarum sieboldii* | 119.03 | 30.37 |
| Host*:Asarum sieboldii* | 117.40 | 30.05 |
| Host*:Asarum sieboldii* | 118.42 | 29.87 |
| Host*:Asarum sieboldii* | 115.94 | 31.73 |
| Host*:Asarum sieboldii* | 119.41 | 30.16 |
| Host*:Asarum sieboldii* | 119.50 | 30.21 |
| Host*:Asarum sieboldii* | 119.28 | 28.60 |
| Host*:Asarum sieboldii* | 119.22 | 30.17 |
| Host*:Asarum sieboldii* | 119.70 | 29.80 |
| Host*:Asarum sieboldii* | 115.31 | 28.89 |
| Host*:Asarum sieboldii* | 117.14 | 27.84 |
| Host*:Asarum sieboldii* | 114.93 | 25.85 |
| Host*:Asarum sieboldii* | 111.86 | 33.05 |
| Host*:Asarum sieboldii* | 111.12 | 32.53 |
| Host*:Asarum sieboldii* | 108.59 | 33.79 |
| Host*:Asarum sieboldii* | 121.79 | 29.82 |
| Host*:Asarum sieboldii* | 107.76 | 34.28 |
| Host*:Asarum sieboldii* | 109.37 | 32.39 |
| Host*:Asarum sieboldii* | 109.14 | 33.95 |
| Host*:Asarum sieboldii* | 107.53 | 33.81 |
| Host*:Asarum sieboldii* | 104.25 | 30.58 |
| Host*:Asarum sieboldii* | 108.04 | 32.09 |
| Host*:Asarum sieboldii* | 113.12 | 36.20 |
