# Supplementary material for: Range Shifts of the Endangered Luehdorfia chinensis chinensis (Lepidoptera, Papilionidae) and Its Specific Hosts in China Under Climate Change
Source: Ecol Evol. 2025 Aug 23;15(8):e72057. doi: 10.1002/ece3.72057 (PMC12374071; doi:10.1002/ece3.72057)
Supplement: Supplementary file 3 — Table S2: Dynamics of the projected suitable habitats of Luehdorfia chinensis chinensis and its two hosts under different climate scenarios. [file ECE3-15-e72057-s002.docx]

**Supplementary Table 2.** Dynamics of the projected suitable habitats of *Luehdorfia chinensis chinensis* and its two hosts under different climate scenarios.

| **Climate scenarios** | | **Low suitable area** | | **Moderately suitable area** | | **Highly suitable area** | | **Total suitable area** | |
| --- | --- | --- | --- | --- | --- | --- | --- | --- | --- |
|  |  | (×10^3^ km^2^） | % | (×10^3^ km^2^） | % | (×10^3^ km^2^） | % | (×10^3^ km^2^） | % |
| **Butterfly: *L.c. chinensis*** | | | | | | | | | |
| Current | | 679.90 | 7.09 | 344.19 | 3.59 | 122.43 | 1.28 | 1146.52 | 11.95 |
| 2041-2060 | SSP1-2.6 | 689.59 | 7.19 | 277.47 | 2.89 | 145.73 | 1.52 | 1112.79 | 11.60 |
|  | SSP2-4.5 | 856.96 | 8.93 | 391.49 | 4.08 | 144.06 | 1.50 | 1392.51 | 14.51 |
|  | SSP5-8.5 | 651.46 | 6.79 | 366.01 | 3.81 | 121.63 | 1.27 | 1139.10 | 11.87 |
| 2061-2080 | SSP1-2.6 | 773.40 | 8.06 | 268.78 | 2.80 | 206.72 | 2.15 | 1248.90 | 13.02 |
|  | SSP2-4.5 | 692.34 | 7.22 | 336.44 | 3.51 | 186.50 | 1.94 | 1215.29 | 12.67 |
|  | SSP5-8.5 | 849.75 | 8.86 | 361.07 | 3.76 | 132.57 | 1.38 | 1343.39 | 14.00 |
| 2081-2100 | SSP1-2.6 | 744.52 | 7.76 | 295.97 | 3.08 | 158.61 | 1.65 | 1199.10 | 12.50 |
|  | SSP2-4.5 | 740.47 | 7.72 | 338.49 | 3.53 | 179.70 | 1.87 | 1258.66 | 13.12 |
|  | SSP5-8.5 | 768.02 | 8.00 | 371.93 | 3.88 | 143.33 | 1.49 | 1283.28 | 13.38 |
| Mean±SD | | 751.83±69.85 | 7.84±0.73 | 334.18±43.91 | 3.48±0.46 | 157.65±27.81 | 1.64±0.29 | 1243.67±89.98 | 12.96±0.94 |
| **Host: *A. forbesii*** | | | | | | | | | |
| Current | | 672.82 | 7.01 | 594.15 | 6.19 | 444.98 | 4.64 | 1039.13 | 10.83 |
| 2041-2060 | SSP1-2.6 | 717.01 | 7.47 | 669.94 | 6.98 | 420.71 | 4.38 | 1090.65 | 11.36 |
|  | SSP2-4.5 | 648.32 | 6.76 | 656.79 | 6.85 | 497.79 | 5.19 | 1154.58 | 12.04 |
|  | SSP5-8.5 | 773.36 | 8.06 | 714.57 | 7.45 | 444.34 | 4.63 | 1158.91 | 12.08 |
| 2061-2080 | SSP1-2.6 | 693.20 | 7.23 | 748.00 | 7.80 | 361.43 | 3.77 | 1109.43 | 11.57 |
|  | SSP2-4.5 | 674.37 | 7.03 | 746.53 | 7.78 | 418.13 | 4.36 | 1164.66 | 12.14 |
|  | SSP5-8.5 | 761.25 | 7.93 | 690.49 | 7.20 | 435.38 | 4.54 | 1125.87 | 11.74 |
| 2081-2100 | SSP1-2.6 | 684.52 | 7.13 | 682.22 | 7.11 | 412.80 | 4.30 | 1095.02 | 11.41 |
|  | SSP2-4.5 | 785.83 | 8.19 | 752.67 | 7.84 | 348.98 | 3.64 | 1101.65 | 11.48 |
|  | SSP5-8.5 | 724.39 | 7.55 | 647.06 | 6.74 | 451.05 | 4.70 | 1098.11 | 11.44 |
| Mean±SD | | 718.03±47.53 | 7.48±0.49 | 700.92±40.97 | 7.31±0.43 | 421.18±45.22 | 4.39±0.47 | 1122.10±29.81 | 11.69±0.31 |
| **Host: *A. sieboldii*** | | | | | | | | | |
| Current | | 658.81 | 6.87 | 1343.27 | 14.00 | 521.45 | 5.43 | 1864.72 | 19.43 |
| 2041-2060 | SSP1-2.6 | 1011.68 | 10.54 | 1083.20 | 11.29 | 476.62 | 4.97 | 1559.82 | 16.26 |
|  | SSP2-4.5 | 848.28 | 8.84 | 1223.77 | 12.75 | 403.68 | 4.21 | 1627.45 | 16.96 |
|  | SSP5-8.5 | 793.17 | 8.27 | 1267.92 | 13.22 | 413.47 | 4.31 | 1681.39 | 17.53 |
| 2061-2080 | SSP1-2.6 | 984.40 | 10.26 | 1270.02 | 13.24 | 327.61 | 3.41 | 1597.63 | 16.65 |
|  | SSP2-4.5 | 803.34 | 8.37 | 1268.61 | 13.22 | 526.57 | 5.49 | 1795.18 | 18.71 |
|  | SSP5-8.5 | 756.66 | 7.89 | 1277.19 | 13.31 | 506.20 | 5.28 | 1783.39 | 18.59 |
| 2081-2100 | SSP1-2.6 | 790.32 | 8.24 | 1323.53 | 13.79 | 445.74 | 4.65 | 1769.27 | 18.44 |
|  | SSP2-4.5 | 722.99 | 7.54 | 1298.07 | 13.53 | 445.77 | 4.65 | 1743.84 | 18.18 |
|  | SSP5-8.5 | 712.81 | 7.43 | 1225.42 | 12.77 | 595.18 | 6.20 | 1820.60 | 18.97 |
| Mean±SD | | 824.85±100.64 | 8.60±1.11 | 1248.63±65.51 | 13.01±0.72 | 460.09±73.32 | 4.79±0.81 | 1708.73±89.65 | 17.97±0.95 |

Note: Mean±SD is the average of the suitable habitat areas of each species under nine future climate scenarios.
